# Between Heritage Conservation and Forensic Science: An Analytical Study of Personal Items Found in Mass Graves of the Francoism (1939–1956) (Spain)

**DOI:** 10.3390/molecules30132783

**Published:** 2025-06-27

**Authors:** María Teresa Doménech-Carbó, Trinidad Pasíes Oviedo, Ramón Canal Roca, Janire Múgica Mestanza

**Affiliations:** 1 Instituto de Restauración del Patrimonio, Universitat Politècnica de València, Camí de Vera 14, 46022 València, Spain; janire.mugica@gmail.com; 2 Museu de Prehistòria de València, Carrer de la Corona, 36, 46003 València, Spain; trini.pasies@dival.es (T.P.O.); ramon.canal@dival.es (R.C.R.)

**Keywords:** forensic science, conservation of heritage, FTIR spectroscopy, FESEM–EDX, light microscopy, XRD, adipocere, scholzite

## Abstract

This article describes the case of the personal items found in common graves dated between 1939 and 1956 after the Spanish Civil War (1936–1939), located in Paterna’s cemetery (Spain). It was important in this study to know the state of the conservation of the objects and to obtain clues about their origin and use just as in a forensic study. This would allow the moral restitution of the historical memory of the victims of the war conflict. The multi-technique strategy has included light and electron microscopy, infrared spectroscopy and X-ray diffraction. Materials of the early 20th century used in pencil sharpeners, glasses, cutlery, lighters, rings, and buttons or medications contained in small bottles and boxes have been identified and have enabled the lives of their owners to be reconstructed during their imprisonment and execution. All these objects exhibited a thin layer of adipocere, a well-known compound in forensic science formed during the decomposition of human and animal corpses. Interestingly, rare corrosion processes have been identified in two of the objects analyzed, which are linked to their proximity to the decomposing corpses of the deceased. Copper sulfides and/or sulfates have been identified in the lighter, and scholzite, a zinc and calcium phosphate, has been identified in the glasses.

## 1. Introduction

**Figure 1:**
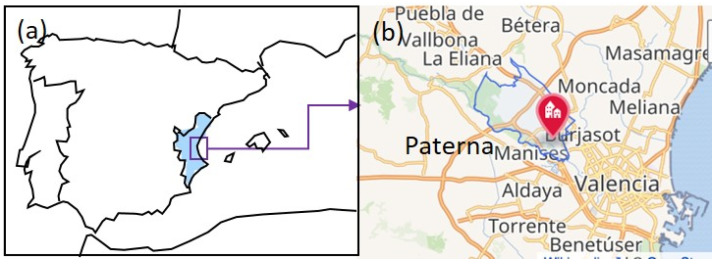
(**a**) Map of Spain; in blue is marked the Valencian Community; (**b**) detail of the map showing the area covered by the Paterna municipality and the town (red point). Adapted from URL: https://www.google.es/maps (accessed on 1 June 2025).

The conservation of cultural heritage usually returns to a distant past and involves archaeological remains and works of art. However, researchers dedicated to the examination and analysis of cultural heritage must sometimes resort to forensic science to obtain clues about the causes and mechanisms that generate the alterations identified in the objects and their origin and use. This is the case for the clothes and personal objects exhumed in several mass graves dated between 1939 and 1956, located in the cemetery of Paterna (Valencia, Spain) [1] (Figure 1).

At the end of the Spanish Civil War (1936–1939), many civilians were retaliated against by Francisco Franco’s government. Many of them were imprisoned, and after a summary trial, they were executed and buried in mass graves without having been afforded the guarantees of a fair trial. In 2012, a systematic and scientific exhumation and identification works began on the remains of people buried in mass graves by an interdisciplinary team formed by archaeologists, anthropologists, historians, restorers, chemists, biologists, forensic scientists, etc. These works were carried out within the legal framework provided by a series of laws and decrees approved in the Spanish parliament and the Valencian government to restore the historical memory of the victims of the war conflict. These laws and decrees regulate how to proceed with the exhumed human remains and their belongings [2,3,4,5].

**Figure 2:**
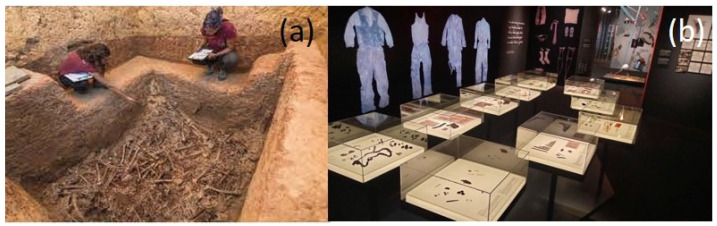
(**a**) The archaeologist Eulàlia Diaz (left) and the anthropologist Gemma López (right), making field work in the mass grave 112 of the Cemetery of Paterna. Courtesy by the photography author: Eloy Ariza—Asociación Científica ArqueoAntro; (**b**) the temporary exhibition held in the Museu de Prehistoria de Valencia: Courtesy by the Archivo SIP del Museu de Prehistòria de València.

The execution of 2237 victims by the defenders of the Franco regime began just four days after the end of the civil war in 1939, and it did not end until seventeen years later, in 1956. The executions took place in Paterna, a town near Valencia (Spain). Once processed, the prisoners were transferred from the prison to the cemetery and were shot at the Terrer, a wall located 500 m from the cemetery. The victims were deposited in the so-called common or mass graves of Paterna. These were up to one hundred square pits that measured 2 and 2.5 m on each side and 1 to 6 m deep (Figure 2a). The bodies were directly thrown into the graves without any care, so they appeared in piles. The condemned men and women, who were taken out of prison in groups to be shot, constituted a saca (sack). The different sacas stacked in the pit can be well distinguished because each was covered with a layer of quicklime. The entire process passed quickly in such a way that the deceased were dressed in the street clothes they were wearing at the moment of the execution. The executors did not bother to take them the street clothes and belongings they were carrying at that time in their pockets. The Valencian Museum of Ethnology (l’ETNO), which custodies the collection, and the Prehistory Museum of Valencia, in collaboration with the Universitat Politècnica de València, undertook the task of restoring the clothes and personal objects of the deceased identified through forensic procedures to creating an exhibition project [6,7,8,9,10,11,12] to pay tribute to the victims. On 5 July 2023, the temporary exhibition “Archaeology of memory. The Paterna graves” was inaugurated by the Prehistory Museum of Valencia (Figure 2b). This temporary exhibition was part of the “The Graves of Francoism: Archaeology, Anthropology and Memory” project co-organized with L’ETNO and the Department of Democratic Memory of the Valencia Provincial Council [13,14,15].

This research was challenging for two reasons. Firstly, due to the great variety of both organic and inorganic materials. Additionally, the personal items of Paterna’s mass graves constitute a peculiar case study due to the particular state of preservation in which they were found, given the aggressive conditions of the burial site. The layers of quicklime between each saca of stacked bodies must have significantly increased the pH of the soil, at least for a time, until the moisture and the rains infiltrated into the ground and reduced the alkalizing effect of the lime that was transformed into calcium carbonate. Another unique aspect of this case study was the presence of a gray–whitish and waxy substance that permeated all objects, especially clothing and other belongings made of organic materials such as rope or wood. The analyses carried out in the project confirmed the hypothesis on the substance’s identity. It was adipocere formed on the corpses by saponification of the fats during the decomposition process of the deceased, which diffused to the clothes and objects in the pockets. In forensic science, the formation of adipoceres is an alteration mechanism that the buried bodies of animals and humans undergo frequently [16,17,18,19,20,21,22,23,24,25]. The adipoceres make it challenging to identify the original components of the objects as they interfere during the analyses. Therefore, the transfer of knowledge from forensic science to archaeology and heritage conservation regarding sample collection, appropriate analytical workflows, and standardized analytical protocols could be beneficial [22].

The large number of samples and the great variety of materials that have constituted the objective of this restoration project have required the application of different instrumental techniques following a multi-technique strategy. Gas chromatography–mass spectrometry (GC–MS) has been widely used for analyzing organic materials because of its high sensitivity and ability to separate and identify the chemical compounds of a polymeric or complex mixture of materials. Nevertheless, this technique is time-consuming as it requires pretreatments of solid samples such as hydrolysis and derivatization. As an alternative, Fourier Transform Infrared Spectroscopy (FTIR) has seen increasing application in heritage science, being comparatively fast and easy to use as none-to-minimal sample preparation is required [26]. These characteristics are essential when many samples with various compositions must be identified. For this reason, this is one of the preferred instrumental techniques used in forensic practice [27]. Optical and scanning electron microscopy techniques (OM, SEM, and field emission FESEM) are also valuable for providing morphological data and elemental composition coupled with an X-ray microanalysis (EDX) system. The minimal amount and the easy and simple preparation of samples required have converted these instrumental techniques into excellent tools for routine analysis in the heritage and forensic sectors. X-ray diffraction (XRD) has also been used for studying the soil composition of the graves.

The analytical chemical study carried out to characterize the personal objects recovered from the Paterna graves is presented in this contribution. Through a multi-technique strategy, it has been possible to answer three of the main questions that the team of historians and restorers raised: From the perspective of the conservation of cultural heritage, what materials were the objects made from? Moreover, what alterations do the objects present? From the perspective of forensic sciences, what is the meaning and function of the belongings for their owners?

**Figure 3:**
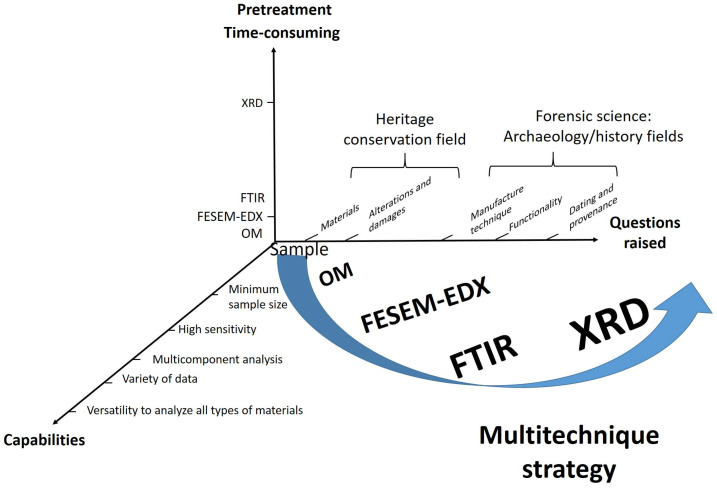
Flowchart illustrating the metastructure of the experimental procedure implemented.

Figure 3 shows a flowchart illustrating the meta-structure of the experimental procedure implemented. The analytical study included the previously indicated instrumental techniques OM, FESEM–EDX, FTIR, and XRD. The multi-technique strategy was based on three criteria that allowed for the selection of analytical techniques and their sequencing in the experimental procedure. The first criterion refers to the minimum requirements that instrumental techniques must meet to be applied to cultural goods (minimum sample size and high sensitivity) and their capabilities that can add value to the analytical process, such as the versatility to analyze materials of all types present in the same sample, performance as a multi-component technique, and capacity to simultaneously provide data of different types (morphological, compositional, and structural). These capabilities determine the qualitative information and quantitative data to be obtained, which constitute the second criterion. These data are necessary to achieve the objectives proposed in the research. The objectives respond to the issues raised on both levels: the knowledge of the personal items in the strict field of the conservation of cultural heritage and the archaeology/history, the latter falling within the stricter scope of forensic science. The third criterion refers to the sample pretreatment requirements of each instrumental technique. While all are non-destructive, the preparation procedure varies from direct sample examination in OM and FESEM–EDX to milling in FTIR and XRD to the more time-consuming cross-section preparation. Analysis time has been another factor to consider, leading to XRD being placed last in the operational sequence and restricted to a small number of soil samples. It is worth noting that most of the conclusions reached about the composition, state of preservation, and functionality of the personal belongings were only possible by the synergies generated by the combined use of the instrumental techniques selected in an appropriate sequence (see Table S1 provided as Supplementary Electronic Material).

## 2. Results

### 2.1. The Collection of Personal Items of the Mass Graves of Paterna

**Figure 4:**
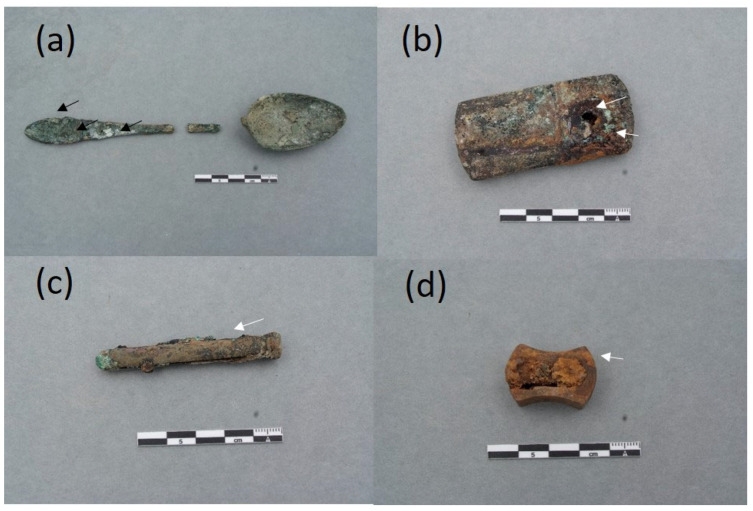
Objects and sampling points. (**a**) Spoon ETN006, deceased 43, mass grave 115; (**b**) cigarette lighter ETN009, deceased 131, mass grave 115; (**c**) pencil extender ETN010, deceased 144, mass grave 115; (**d**) pencil sharpener ETN012, deceased 107, mass grave 115. Photographs by courtesy of the Archivo SIP del Museu de Prehistòria de València. Arrows highlight the sampling points in the artefacts. Marks in the scalebars correspond to 1 cm.

**Figure 5:**
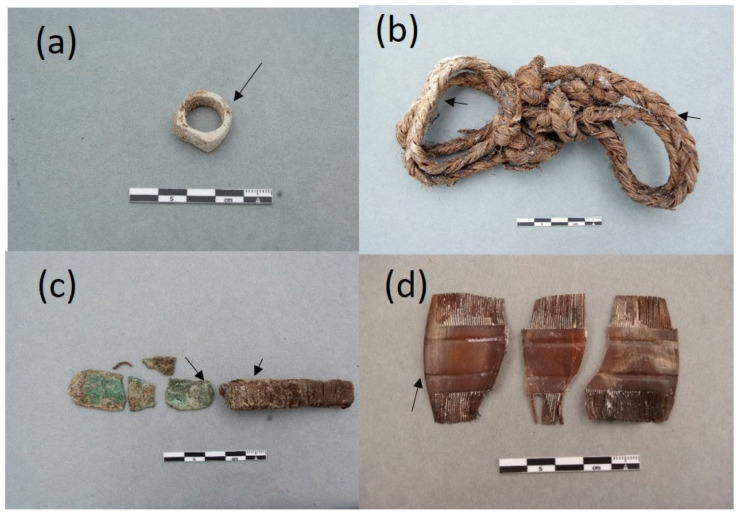
Objects and sampling points. (**a**) Stone ring ETN053, deceased 28, mass grave 94; (**b**) rope handcuffs ETN045, deceased 92 identified by DNA analysis as Mr. José Peiró I Grau, mass grave 112; (**c**) knife ETN048, deceased 15, mass grave 100; (**d**) nit comb ETN060, deceased 66 identified by DNA analysis as Mr. Juan Bautista Solanes i González, mass grave 112. Photographs by courtesy of the Archivo SIP del Museu de Prehistòria de València. Arrows highlight the sampling points in the artefacts. Marks in the scalebars correspond to 1 cm.

**Figure 6:**
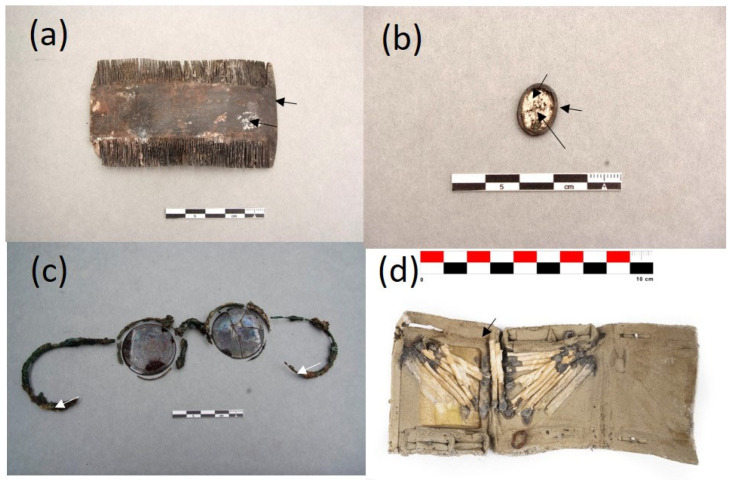
Objects and sampling points. (**a**) Nit comb ETN058, deceased 95, mass grave 112; (**b**) medal ETN067, deceased 68, mass grave 128; (**c**) spectacles frame ETN073, deceased 83, mass grave 111; photographs (**a**–**c**) by courtesy of Archivo SIP del Museu de Prehistòria de València; (**d**) cigarette paper in a matchbox F111-141, deceased 141, identified by DNA analysis as Mr. Vicente Orti, mass grave 111. Courtesy by the photography author: Eloy Ariza—Asociación Científica ArqueoAntro. Arrows highlight the sampling points in the artefacts. Marks in the scalebars correspond to 1 cm.

**Figure 7:**
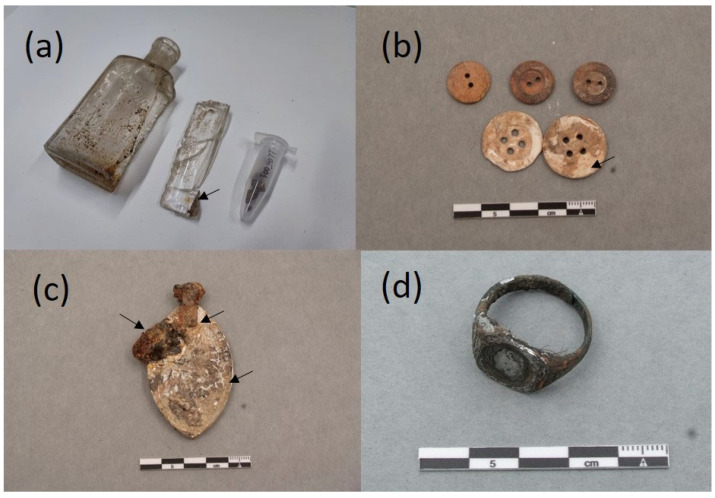
Objects and sampling points. (**a**) Organic remains inside a glass bottle with the inscription “Naranjil Purgante ideal” LLUE001, deceased 14, identified by DNA analysis as Mr. Manuel Lluesma i Masiá, cemetery niche 645; (**b**) button MCP001, deceased 19, mass grave 114; (**c**) devotional medallion with rests of a rope drawstring and textile fibers from clothes adhered MCP029, deceased 176, mass grave 114; (**d**) metal ring ETN033, deceased 73, mass grave 112. Photographs by courtesy of the Archivo SIP del Museu de Prehistòria de València. Arrows highlight the sampling points in the artefacts. Marks in the scalebars correspond to 1 cm.

The collection of objects presented in this paper is a representative sample of the set of belongings analyzed. It is composed of 16 items that are shown in Figure 4, Figure 5, Figure 6 and Figure 7. A detailed description of the items is summarized in Table S1.

### 2.2. Identification of Materials

#### 2.2.1. Elemental Composition of Metallic Objects

Several personal items of metallic nature were identified by its elemental composition, which was determined by using FESEM–EDX. Table 1 summarizes the results obtained.

In the samples from the spoon ETN006 and the knife ETN048, copper, zinc, and nickel were identified as the main components. A quaternary brass alloy was identified in the sample ETN009-1 excised from the upper layer of the cigarette lighter case. The major elements in the brass are copper and zinc, accompanied by smaller amounts of lead and tin found in some spot analyses. Iron was associated with the inner layers of the case and other lighter pieces, such as the hinges, that could be made of steel (sample ETN009-2). Copper, zinc, and silver were the elements identified in the sample ETN010 from the pencil extender. These metals are associated with a binary brass alloy with silver plating. Aluminum was identified in the sample MCP029 from a devotional from a medallion, and silver–copper alloy was used in the plating (ETN033-1) of a ring with iron body (ETN033-2). The X-ray spectra acquired in the sample ETN067 from the medal metal mount exhibited emission lines of copper, zinc, and lead associated with a ternary brass. In the X-ray spectrum of a second sample excised in the central part of the medal, intense sulfur and barium emission lines were identified associated with the white pigment barite. Traces of cobalt suggest that a blue cobalt pigment was also used in the medal decoration.

#### 2.2.2. Mineralogical and Micromorphological Characterization of Inorganic Materials

**Figure 8:**
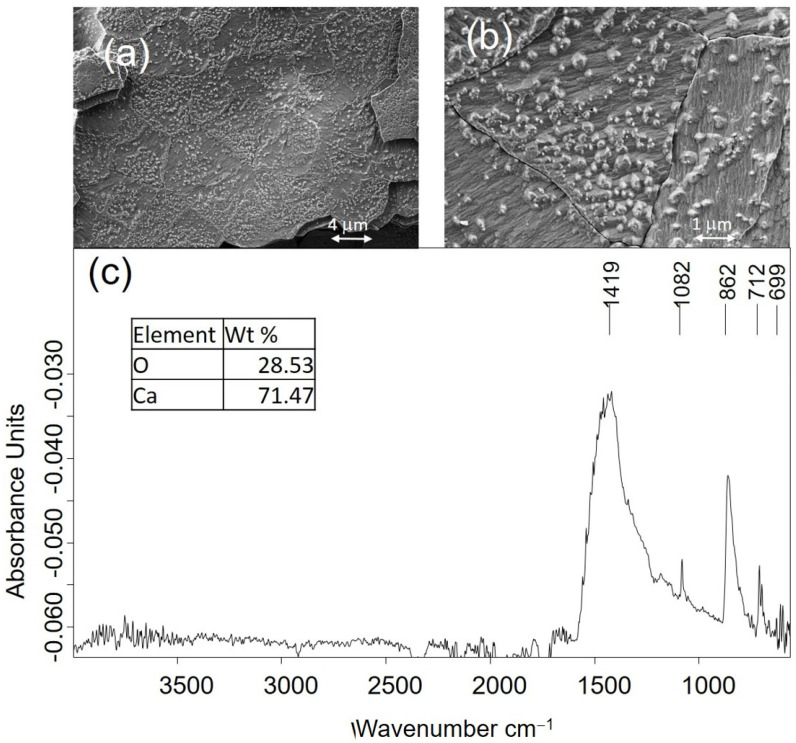
(**a**) Secondary electron images of the sample MCP001; (**b**) detail of the aragonite platelets and mineral bridges; (**c**) IR absorption spectrum. The inset shows the elemental composition obtained using FESEM–EDX.

Some of the materials that compose the objects were identified directly by optical and electron microscopy examination. That morphological identification was further confirmed from their elemental and molecular analysis using FESEM–EDX and FTIR spectroscopy. For example, the secondary electron images obtained in a small sample of the item MCP001, a shirt button, are shown in Figure 8. The button’s nacre (mother-of-pearl) material is easily recognized by its microscopical morphology. That is an organic–inorganic composite material of biogenic origin produced by some mollusks as an inner shell layer. The nanoscopic and polygonal platelets that form the laminas of the nacre “brick-and-mortar” structure are seen in Figure 8a. Figure 8b shows the mineral bridges and the micro-roughness of the surface platelets. Figure 8c shows the IR absorption spectrum acquired in this sample. In the inset, the chemical composition obtained by FESEM–EDX is shown, which suggests the calcium carbonate composition of this biomineral. The position of the IR bands confirms that the nacre is mainly composed of the mineralogical species aragonite (CO_3_ asymmetric stretching ν_3_ at 1419 cm^−1^; CO_3_ symmetric stretching ν_1_ at 1082cm^−1^; CO_3_ asymmetric deformation ν_2_ at 862cm^−1^; and CO_3_ symmetric deformation ν_4_ at 712 and 699cm^−1^) [28].

**Figure 9:**
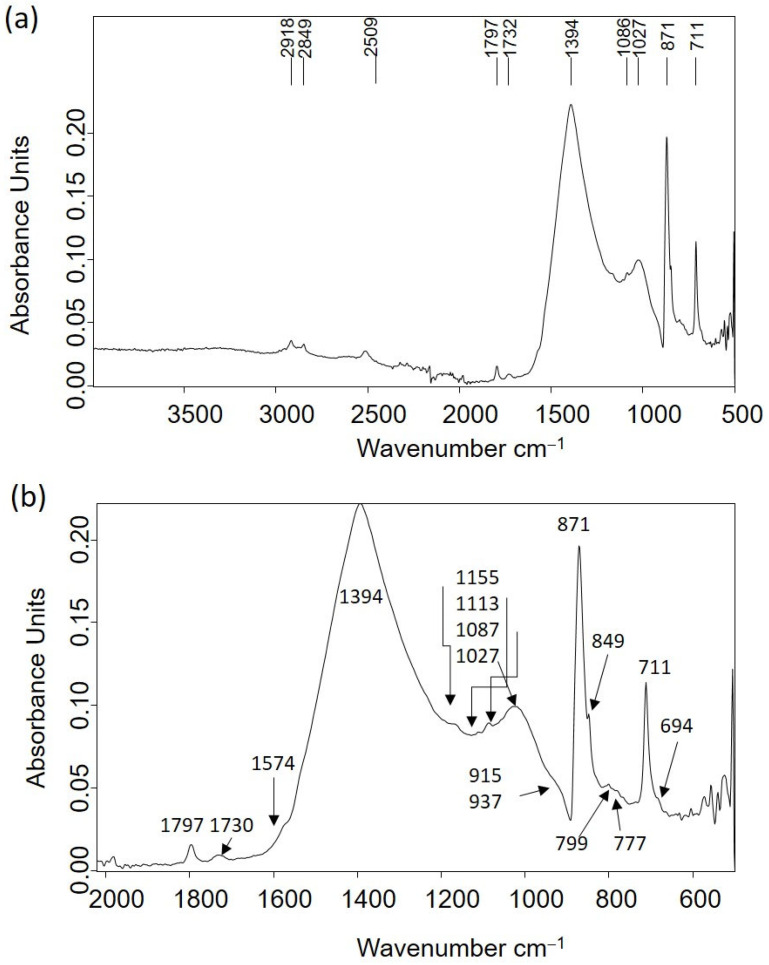
(**a**) IR absorption spectrum of sample ETN053 excised from a limestone ring; (**b**) detail of the IR spectrum in the 600–2000 cm^−1^ region.

Other materials of an inorganic nature that compose several personal items were identified from the IR spectra acquired in the samples excised from these items. Table 2 shows the leading IR absorption bands of analytical interest and their assignation to chemical compounds that constitute the ring ETN053. The IR absorption spectrum is shown in Figure 9a. Limestone, mainly composed of calcite with minor amounts of clays, quartz, and organic matter of lipid characteristics, is the material that composes the ring. In Figure 9b, several overlapped bands appear in the 1600–600 cm^−1^ region. Remarkably, the band at 1574 cm^−1^ is ascribed to calcium carboxylate complexes [29].

#### 2.2.3. Molecular Structure and Micromorphological Characterization of Organic Materials

**Figure 10:**
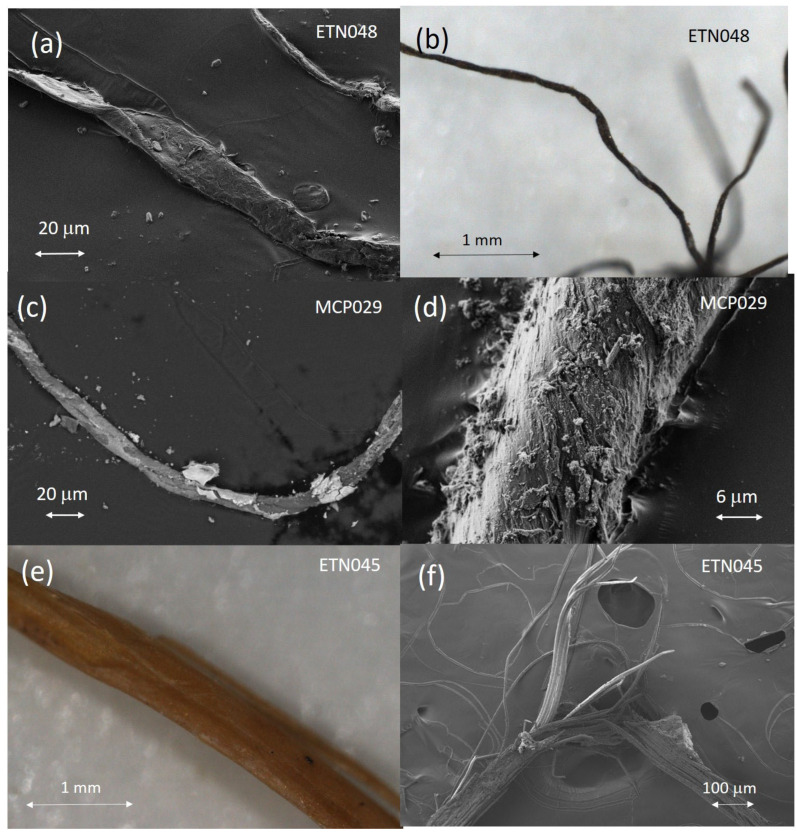
(**a**) Secondary electron images of fibers that formed the thread wound around the handle of the knife ETN048; (**b**) microphotograph taken in sample ETN048; (**c**) backscattered electron images of cotton fibers of the cord drawstring attached to the devotional medallion MCP029; (**d**) detail of the micromorphology of the MCP029 fibers; (**e**) microphotograph taken in rope handcuffs fibers of the sample ETN045; and (**f**) secondary electron image of fibrils of the sample ETN045.

The natural fibers found in the personal items ETN048 and MCP029 could be identified by their characteristic micromorphology observed in the microphotographs and secondary electron images shown in Figure 10. The fibers that formed the yarns wound around the tag of the knife ETN048 presented ribbons with convolution and a kidney shape in the cross-section characteristic of cotton (Figure 10a,b). The average fiber diameter measured is 21 μm. An IR absorption spectrum acquired in this sample (Figure S1) allowed us to recognize a proteinaceous substance, which is associated with some binding substance used for adhering the fibers to the metallic tang of the knife. This material was characterized by IR bands ascribed to amide A (NH- stretching) at 3264 cm^−1^; amide B (NH- stretching) at 3072 cm^−1^; CH_2_ and CH_3_ stretching vibrations at 2956, 2922, and 2852 cm^−1^; amide I band at 1625 cm^−1^; amide II band at 1539 cm^−1^; amide III band and bending vibrations of the CH bonds of methyl and methylene groups at 1451 and 1399–90 cm^−1^; and stretching vibrations of triglyceride ester linkages at 1232, 1159, and 1033 cm^−1^. Cotton fibers formed the cord drawstring attached to the devotional medallion MCP029 (Figure 10c). This image also shows a thin crust of minerals and corrosion products covering the fibers. Figure 10d shows in detail the fibril micromorphology typical of cotton fibers and the high degree of deterioration due to the aggressive burial environment. Figure 10e shows an image of the esparto fibers that compose the rope handcuffs ETN045 characterized by the bundles of cellulosic fibrils (Figure 10f).

**Figure 11:**
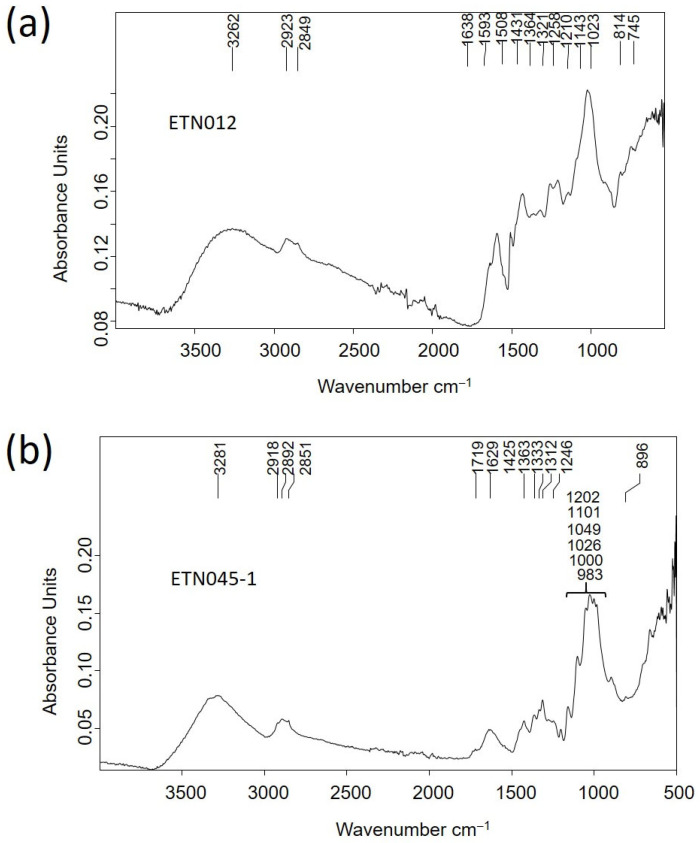
IR absorption spectra acquired in samples excised of the personal items: (**a**) sharpener ETN012; (**b**) Esparto grass rope handcuffs ETN045-1.

The materials that compose the pencil sharpener ETN012 and the rope handcuffs ETN045 were identified as wood and vegetal fibers of esparto, respectively, using FTIR spectroscopy. The IR spectrum of sample ETN012 (Figure 11a) exhibited bands characteristic of wood ascribed to vibrations of H-bonded to OH-groups intermolecular in cellulose at 3262 cm^−1^, C-H stretch in methyl and methylene groups at 2923 cm^−1^, CH_2_ asymmetric stretching and methoxyl C-H stretching at 2849 cm^−1^, adsorbed water at 1638 cm^−1^, aromatic skeletal vibrations plus C=O stretch at 1593 cm^−1^, overlapped HOC bending, aromatic skeletal vibration combined with C-H in plane deformation and CH_2_ scissoring at 1431 cm^−1^, overlapped aliphatic C-H stretch in CH_3_, symmetric C-H bending from the methoxy group and OH in plane deformation vibration at 1364 cm^−1^, CH_2_ rocking vibration at 1321 cm^−1^, G ring plus C=O stretch at 1258 cm^−1^, OH in plane deformation at 1210 cm^−1^, aromatic C-H in plane deformation typical for G units, whereby G condensed >G etherified, at 1143 cm^−1^, overlapped asymmetric in-phase ring stretching, aromatic in-plane deformation plus C-O deformation in primary alcohols plus C=O stretch and C_alkyl_-O ether vibration in guaiacol at 1023 cm^−1^, vibrations of glucomannan at 814 cm^−1^, and rocking vibration of cellulose at 745 cm^−1^ [31,32].

The rope handcuff fibers of the sample ETN045 were identified as esparto by their characteristic micromorphology observed in the optical microphotographs and secondary electron images obtained by OM and FESEM (Figure 10e,f). These fibers exhibited in the IR spectrum (Figure 11b) characteristic bands associated with H-bonded to OH stretching vibrations at 3281 cm^−1^, CH_2_ asymmetric stretching at 2921 cm^−1^, CH_2_ asymmetric stretching at 2892 cm^−1^, CH_2_ symmetric stretching vibration at 2851 cm^−1^, C=O stretching of oxidized cellulose and carbonyl and carboxyl stretching in rosin at 1719 cm^−1^, absorbed water in cellulose at 1629 cm^−1^, CH_2_ bending and asymmetric C-O stretching in cellulose at 1425 cm^−1^, CH bending of cellulose at 1363 cm^−1^, OH in-plane bending in cellulose at 1333 cm^−1^, CH wagging of cellulose at 1312 cm^−1^, OH out-of-plane bending in cellulose at 1246 cm^−1^, OH in-plane bending in cellulose at 1202 cm^−1^, asymmetric in-plane ring stretching in β-glycoside (C_1_-O-C_4_) in cellulose at 1101 cm^−1^, C-O stretching in second alcoholic groups in cellulose at 1049 cm^−1^, C-O stretching in first alcoholic groups in cellulose at 1026 cm^−1^, C-O and C-H stretching at 1000 cm^−1^ and asymmetric out-of-phase ring stretching in β-glycoside (C_1_-O-C_4_) bond in cellulose at 896 cm^−1^ [33].

**Figure 12:**
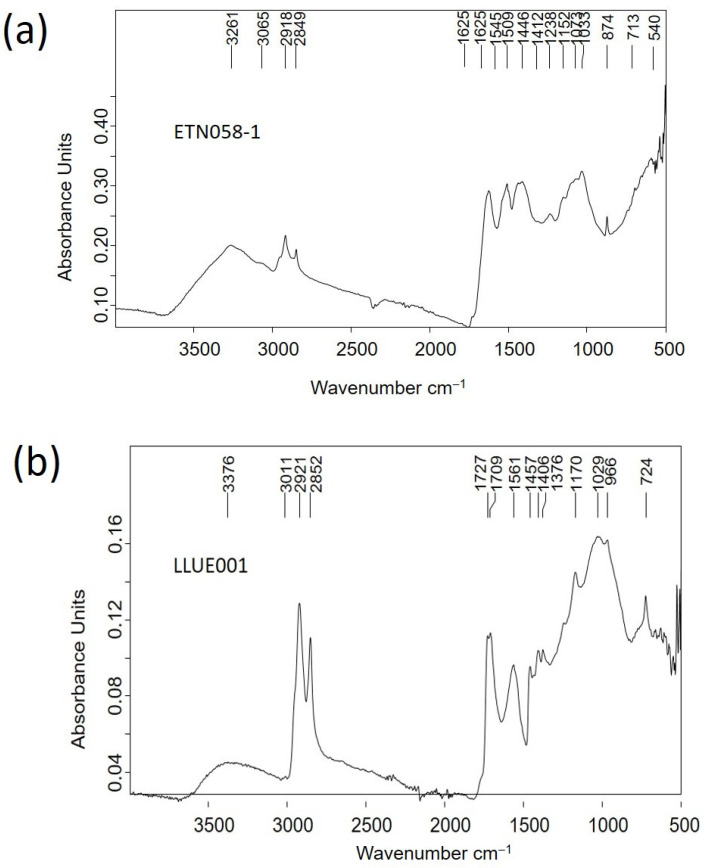
IR absorption spectra acquired in samples excised of the personal items: (**a**) nit comb ETN058-1; (**b**) organic remains inside a glass bottle with the inscription “Naranjil Purgante ideal” LLUE001.

Similarly, a casein-formaldehyde resin (galalith) was the main component identified in the items ETN058, ETN060, and ETN073 (Figure 12a, Figures S2 and S3). The IR spectra included bands ascribed to N-H stretching at 3261 cm^−1^, amide B (NH- stretching) at 3065 cm^−1^, aliphatic C-H asymmetric stretching at 2918 and 2956 cm^−1^, aliphatic C-H symmetric stretching at 2874 and 2849 cm^−1^, C=O ester carbonyl band at 1731 cm^−1^, amide I band (C=O stretching amide) at 1626 cm^−1^, amide II band (N-H bending) at 1545 cm^−1^, C-H bending at 1446 cm^−1^, C-N stretching at 1397 cm^−1^, interaction between the NH bending and CN stretching of the C-N-H group at 1238 cm^−1^, and O-C-C stretching at 1074 cm^−1^ [34]. Bands at 1412, 874, and 713 cm^−1^ are assigned to calcite, the shoulder at 1565 and the band at 1515 cm^−1^ are assigned to calcium carboxylates, bands at 1152, 1074, and 1034 cm^−1^ are assigned to quartz and clayey minerals, and the broad and intense band at 540 cm^−1^ is attributed to stretching Fe-O bands in iron-red oxide (see Table 2 for assignments).

Organic remains inside a glass bottle with the inscription “Naranjil Purgante ideal” LLUE001 exhibited characteristic bands of an oil associated with H-bonded OH stretching vibrations at 3376 cm^−1^, alkenyl C=C stretching vibrations at 3011 cm^−1^, CH_2_ asymmetric stretching at 2921 cm^−1^, CH_2_ symmetric stretching at 2852 cm^−1^, C=O asymmetric stretching of ester groups at 1727 cm^−1^, CH_2_ symmetric deformation at 1457 cm^−1^, carboxylic group deformation at 1406 cm^−1^, CH_3_ symmetric deformation at 1376 cm^−1^, C-(C=O)-O stretching of ester groups at 1170 cm^−1^, O-C-C stretching of ester groups at 1029 cm^−1^, -C=CH- out-of-plane bending at 966 cm^−1^, and -CH_2_- asymmetric bending (C-H rocking) at 724 cm^−1^ [35]. C=O asymmetric stretching (*ν*_α_) of fatty monoacid groups is found at 1709 cm^−1^, *ν*_α_ of fatty diacids appear at 1700^sh^ cm^−1^, and *ν*_α_ of calcium carboxylate of fatty mono and diacids are found at 1561, 1549^sh^, and 1509^sh^ cm^−1^ [36] (Figure 12b).

### 2.3. Adipoceres

**Figure 13:**
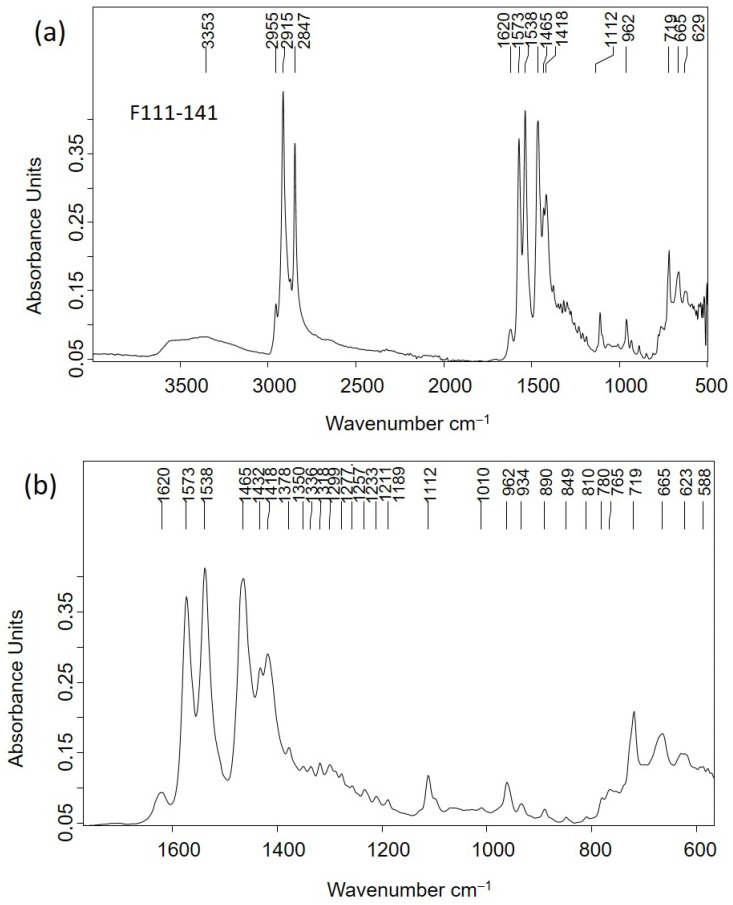
IR absorption spectra acquired in samples excised of the personal items: (**a**) cigarette paper in a matchbox F111-141; (**b**) detail of the 600–1800 cm^−1^ region.

Most of the personal items were impregnated with a white–greyish waxy substance that was associated with a well-known phenomenon in forensic sciences related to a series of specific chemical processes of the decomposition of the fatty matter of human and animal cadavers in burials (see Figure 5a,b,d Figure 6a–d and Figure 7c) [16,17,18,19,20,21,22,23,24,25]. The adipoceres were unambiguously identified by FTIR spectroscopy in all the personal belongings. Three types of adipoceres were identified in the set of samples analyzed that differ in the composition of the compounds that form this complex substance. In most of the personal belongings, the adipoceres identified were characterized by the significant presence of the calcium salts of fatty acids among the compounds that form the mixture (see, as an example, Figure 13a and Figure S4 corresponding to cigarette paper F111-141 and rope handcuffs ETN045). Calcium salts were prevalent due to the easy adsorption of the fatty acids to calcium carbonate in the burial environments and the low solubilities in water [37]. Bands at 3563, 3353, and 1620 cm^−1^ were ascribed to the OH stretching and bending of crystalline water in hydrated calcium salts. The features from the hydrocarbon chains are observed at 2955 and 2915 cm^−1^ (antisymmetric stretching of CH_3_ and CH_2_ groups) and 2874 and 2847^sh^ cm^−1^ (symmetric stretching of CH_3_ and CH_2_ groups). The value of 2915 cm^−1^ for the *ν*_a_ CH_2_ groups points to saturated fatty monoacid salts with chain lengths up to C14:0 [36]. Bands of carboxylate groups are found at 1573 and 1538 cm^−1^ (antisymmetric stretching) and 1465, 1432, and 1418 cm^−1^ (symmetric stretching and *δ* CH_2_ scissoring doublet) characteristic of uni- and bidentate calcium fatty monoacids salts (Figure 13b) [38]. Sub-bands from calcium fatty monoacid salts ascribed to twisting vibration (*τ* CH_2_) are found at 1378, 1350, 1336, 1318, and 1299 cm^−1^. A pattern of bands at 1277, 1257, 1233, 1211, 1205^sh^, and 1189 cm^−1^ is ascribed to wagging vibrations (*ω* CH_2_). These multiplet series are typical of calcium fatty monoacid salts. Stretching vibration of (C-OH) group occurs at 1112 cm^−1^. Unassigned band at 962 cm^−1^ and band at 719 cm^−1^ assigned to rocking vibration (*ρ*) of CH_2_ groups are also observed [36].

**Figure 14:**
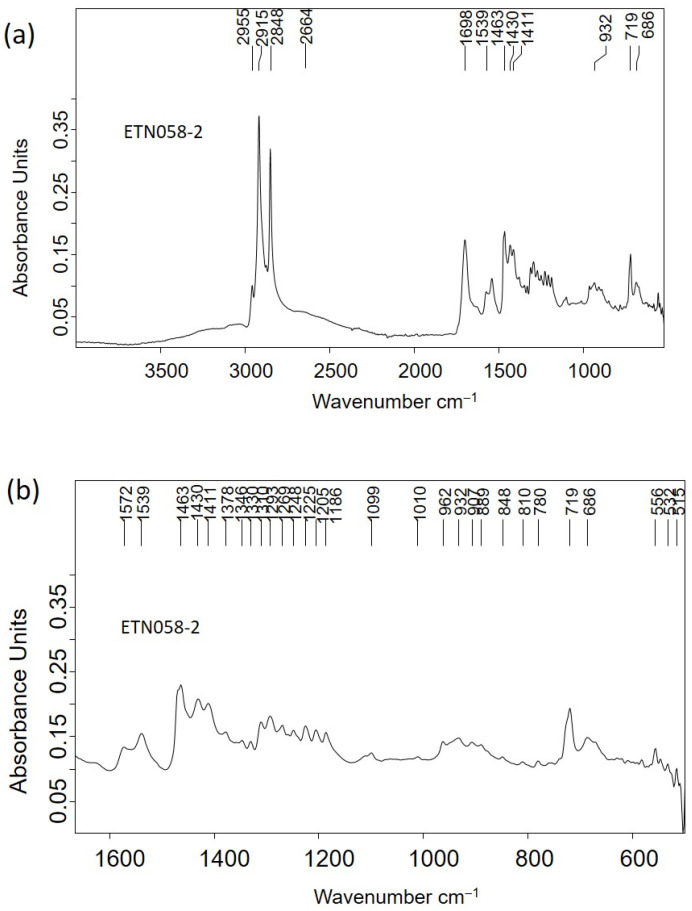
IR absorption spectra acquired in samples excised of the personal items: (**a**) nit comb ETN058; (**b**) detail of the 600–1800 cm^−1^.

Although the spectral profile of the IR spectrum acquired in the nit comb sample ETN058 exhibited the main absorption bands that were previously described, specific differences were found (Figure 14a). In particular, the band at 2664 cm^−1^ is assigned to hydroxyl–carbonyl interactions due to fatty monoacid dimers, and the band at 1698 cm^−1^ is attributed to the C=O stretching vibrations of carboxylic groups. Like in the calcium salts, the *δ* CH_2_ scissoring vibration splits into two components at 1463 and 1411 cm^−1^ and the umbrella vibration at 1378 cm^−1^. The low intensity (Figure 14b) of these bands indicates that the fatty acids in the adipocere have long chains like stearic (octadecanoic) acid (C18:0) or up to that. The twisting vibration is split throughout 1378–1310 cm^−1^, whereas the wagging vibration is found in the range 1310–1186 [39]. According to Filipoulou et al. [36], the double split in the twisting region indicates length chains of C14:0-C18:0. In the wagging region the number of bands that form the multiplet increases with the hydrocarbon chain length following the rule $\omega =\frac{n}{2}-1$, where *n* is the number of carbons in the hydrocarbon chain. Thus, the seven bands identified with two shoulders at 1259 and 1239 cm^−1^ in this region suggest that the main component of this adipocere is palmitic (hexadecenoic) acid (C16:0), accompanied by stearic acid (C18:0) and arachidic (eicosanoic acid) (C20:0). This result is confirmed by the calculated distancing between consecutive bands in the range 18–20 cm^−1^.

The IR spectrum ETN073-2 acquired in a sample of adipoceres excised from the metal spectacles frame is shown in Figure S5. The contact of adipoceres with the parts of the brass surface of the spectacles frame exposed has resulted in the formation of zinc salts of long-chain fatty monoacids, which are recognized by the occurrence of asymmetric stretching vibrations of the Zn-carboxylate group at 1591 (tetranuclear complex) and 1536 cm^−1^, symmetric stretching at 1397 cm^−1^, and doublet at 74^sh^ and 719 cm^−1^ ascribed to the rocking vibration of CH_2_ groups, together with bands at 1464^sh^ and 1454 cm^−1^ assigned to the scissoring vibrations of CH_2_ groups ascribed to zinc salts of C8:0 and C9:0 short-chain fatty diacids [36].

### 2.4. Corrosion Products

**Figure 15:**
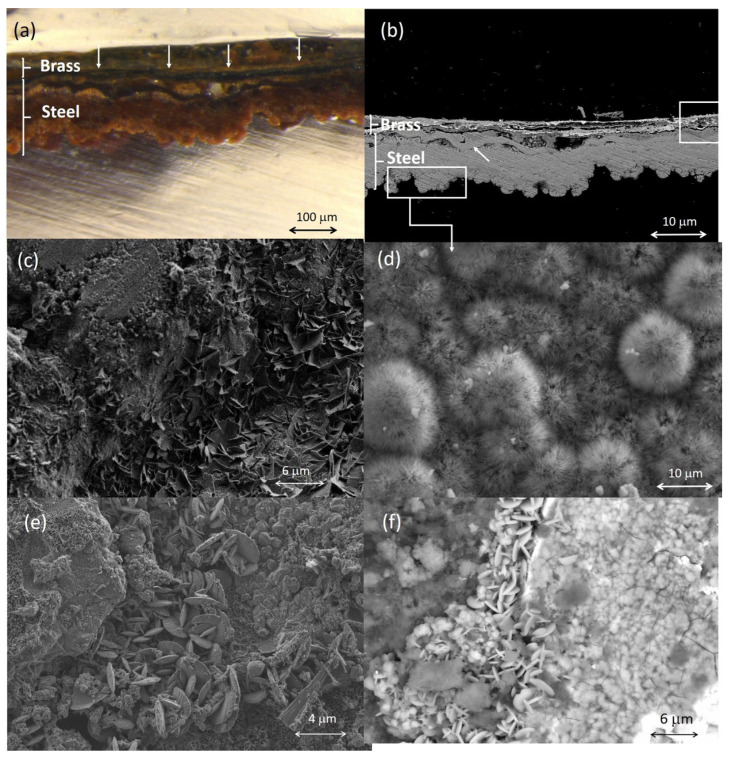
(**a**) Microphotograph of a sample excised from the case of the cigarette lighter ETN009 prepared as cross-section, where the arrows point out the upper surface of the cross-section in the sample; (**b**) backscattered electron image acquired in the cross-section shown in (**a**); detailed analysis of the upper framed area is shown in (**d**); (**c**) secondary electron image of crystals of lepidocrocite in the outer layers; (**d**) backscattered electron image of semicrystalline goethite formed in the inner layer; (**e**) secondary electron image of hydrocerussite crystals; (**f**) backscattered electron image of platy crystals of copper corrosion products.

The electron images complemented with X-ray microanalysis and IR spectra have allowed the identification of some mineral corrosion products formed in certain metallic items by their characteristic micromorphology. Figure 15a,b show the microphotograph and the backscattered electron image acquired in the cross-section of a sample excised from the case of the cigarette lighter ETN009. The case is made with steel covered by a thin brass layer. Both parts show clear signs of alteration. In the outermost layer, the remains of what appears to be the original brass can be seen, forming a thin, highly fragmented sheet dispersed in a mixture of corrosion products and infiltrating exogenous materials that form a layer at ca. 30 µm. Those infiltrations could be favored by the formation of copper corrosion products that produce swelling and increase the porosity of the layer [40,41]. Beneath the brass, the upper part of the steel foil can be seen, with evidence of complete corrosion, which would have caused it to flake off from the inner unaltered steel core (not shown in the image) as result of the advance of the corrosion process. The arrow in Figure 15b indicates a thin (12 µm) band that presents ripple-like dislocations. This particular feature is tentatively associated with a differential elongation of the steel grains in the surface of the foil that could have occurred during the manufacture of the steel plate used to make the lighter case by the cold-rolling process [42]. Figure 15c shows fine-plate crystals of lepidocrocite (g-FeO(OH)) formed in the pores and fissures connected with the external soil. This crystalline material forms flower-like structures [43]. Figure 15d shows the semicrystalline goethite (a-FeO(OH)) formed in the case. The particles exhibit a cotton-ball characteristic morphology [44,45]. Alteration products of the metals that form the brass have also been identified by their characteristic morphology. The rounded platelet-shape of the particles found in the case surface are identified as hydrocerussite (Pb_3_(CO_3_)_2_(OH)_2_) (Figure 15e). Figure 15f shows submicron-sized crystals with a flake-like morphology. EDX analysis of these particles reported molar ratios of O/Cu = 4.2 and S/Cu = 1.1. Analogous crystal micromorphologies have been described for brochantite [46] and posnjankite [40] grown in copper patinas formed by exposition to the atmosphere and are very similar to covellite (CuS) [47].

**Figure 16:**
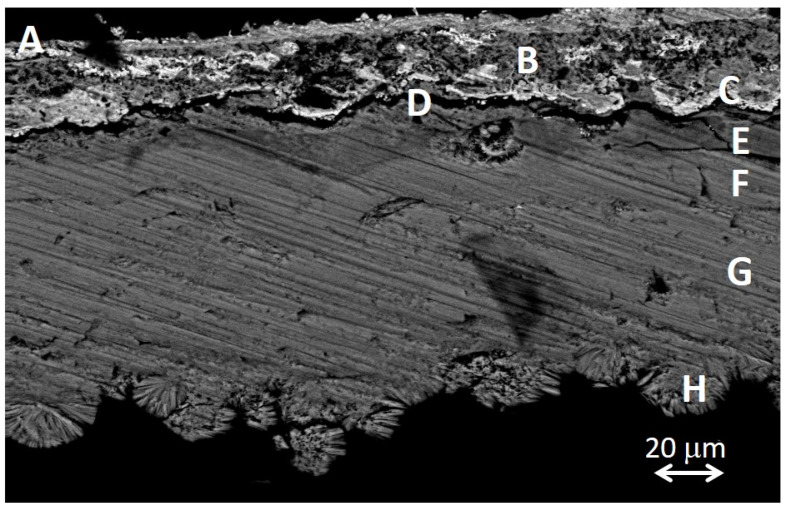
Backscattered electron image acquired in the cross-section of the cigarette lighter ETN009 (see area framed in Figure 15b). A, B and C correspond to the three layers and areas compositionally differentiated in the brass upper cover of the cigarette lighter; D, E, F, G and H correspond to the five layers and areas compositionally differentiated in the steel inner case of the cigarette lighter.

**Figure 17:**
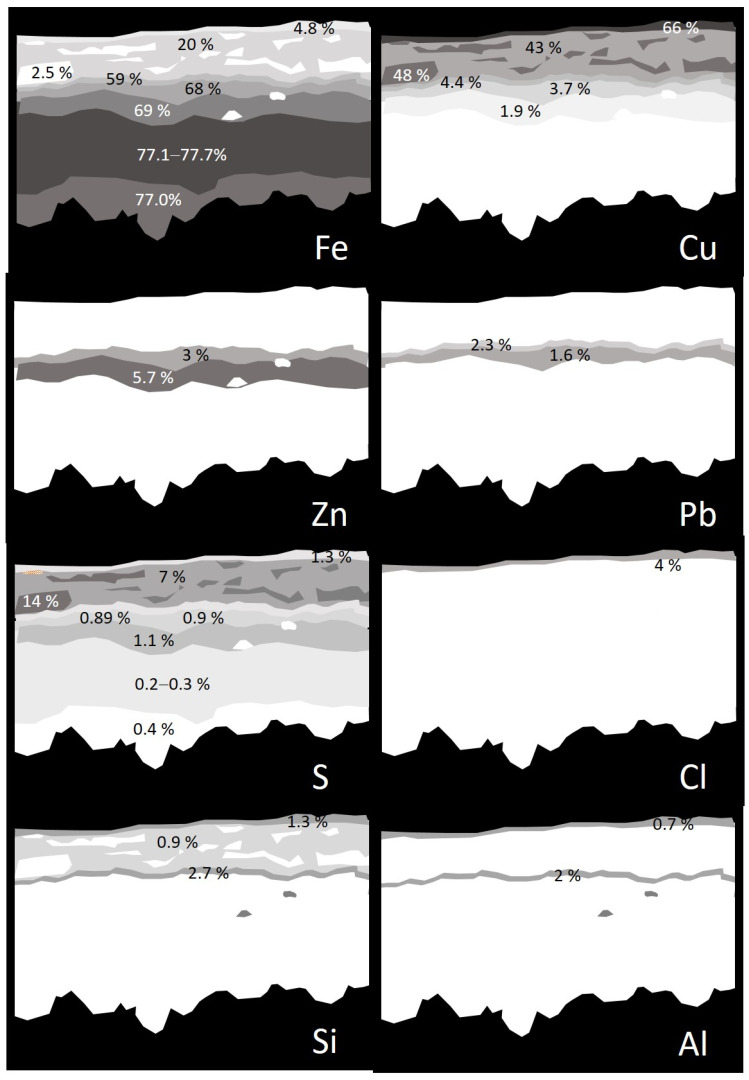
Elemental distribution maps acquired with FESEM–EDX on the backscattered electron image of Figure 16. Values in graphs indicate the weight % composition of the element in each specific area.

Figure 16 shows in detail the complex stratified structure of the corrosion layers of the sample ETN009. The different layers and areas compositionally differentiated have been labeled from A to H. The overall elemental composition of the layers and areas is provided in Table S2. The two-dimensional distribution of the content of the different elements is graphically illustrated in the X-ray maps shown in Figure 17. Oxygen is present in all the layers in concentrations over 22 wt%, indicating that the corrosion process has progressed considerably and no or little unaltered brass or steel still remains. The A to C regions correspond to the brass quaternary alloy Cu-Zn-Pb-Sn, whereas areas D-H are located in the steel layer. Chlorine (~1%) is present in the outermost layer A. Sulfur is present along the cross-section with prevalence in B and C regions, with concentrations of 7.3 and 14.5%, respectively. That indicates that copper sulfate or sulfide species were formed. However, the Cu/S molar ratios calculated by the ZAF method do not allow any conclusion and could suggest that non-stoichiometric compounds are present in these layers. The significant presence of Fe in the outermost layers A to C indicates that the diffusion of Fe ions throughout the brass layer has taken place. Other foreign elements such as Al, K, and Si in the A, B, and D layers confirm that these strata are highly altered regions with easy access to the attacking solution and the exogenous materials. The lower D to H regions correspond to the steel structure of the lighter case. The interphase between the brass cover and the steel case (region D) has the lowest Fe content (59.0%) and some Cu, Zn, and Pb. These elements are also found in areas E and F, suggesting that diffusion from the outermost brass layer has occurred at some extent. The Fe content increases from 59.0% to 77.7% when it passes from layer D to H, maintaining a Fe/O molar ratio of 0.98–1.00. This molar ratio value, around 1, suggests the presence of mixtures of oxides and oxyhydroxides that prevalently approximate the structure of magnetite and goethite [48]. Small amounts of sulfur are present throughout the entire section of the steel sheet, ranging between 0.89 and 1.41 wt% in D-F regions and 0.2–0.39 wt% in the G-H regions.

**Figure 18:**
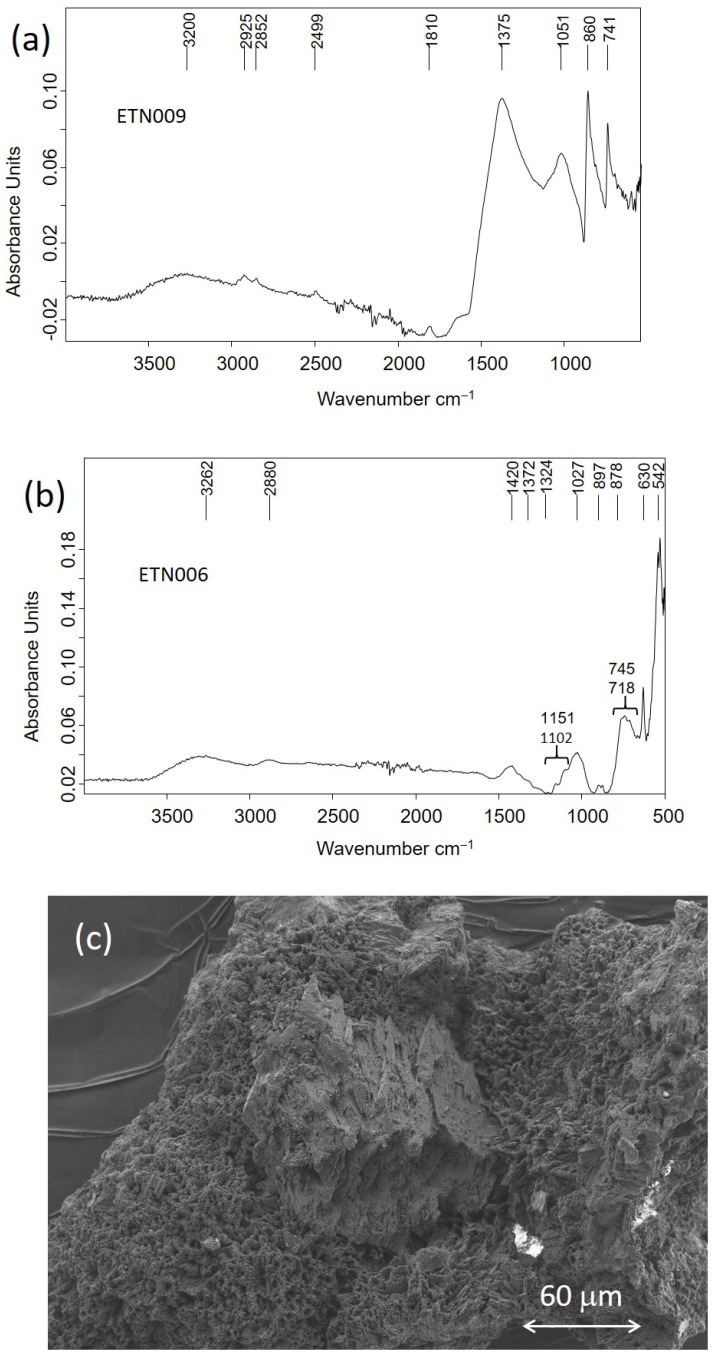
(**a**) IR absorption spectrum of cigarette lighter ETN009; (**b**) spoon ETN006; (**c**) secondary electron image acquired in a sample excised from the corrosion products of the metal spectacles frame ETN073.

A similar cryptocrystalline morphology of hydrocerussite to that shown in Figure 15e was found in the corrosion layers of the pencil extender ETN010. The IR spectra acquired in samples of corrosion products formed on the surface of the metallic objects confirmed that these external corrosion layers were complex mixtures of corrosion salts, sometimes of inorganic–organic hybrid character. That is the case of the corrosion layer formed in the cigarette lighter ETN009 and the spoon ETN006. Figure 18a shows the IR spectrum acquired in the sample ETN009. The absorption bands of malachite CuCO_3_.Cu(OH)_2_ dominate the spectrum, namely, the 2ν_2_ + ν_4_ CO_3_ stretching vibration at 2499 cm^−1^, ν_1_ + ν_4_ CO_3_ stretching vibration at 1810 cm^−1^, ν_3_ CO_3_ asymmetric stretching vibration with maximum at 1375 cm^−1^, ν_2_ asymmetric deformation at 860 cm^−1^ with shoulder at 840 cm^−1^, and ν_4_ symmetric deformation at 741 cm^−1^ [49]. The presence of siderite from the case and calcite from soil particles integrated into the sample cannot be ruled out as they would overlap in the previous bands, given the proximity between the absorption band maxima. OH stretching and deformation bands at 3200 and 1670 cm^−1^, respectively, and broad band at 1051 cm^−1^ associated with asymmetric stretch ν_3_ SO_4_ are ascribed to sulfates [50]. Bands of stretching vibrations of methyl and methylene groups in organic compounds occur at 2925 and 2852 cm^−1^. The IR spectrum acquired in a sample of the corrosion layer formed on the spoon ETN006 surface is shown in Figure 18b. Calcite is identified by the bands at 1420, 878, and 718 cm^−1^; bands of quartz and clays occur at 1151, 1102, and 1027 cm^−1^ (see Table 2 for assignment); hydroxyl stretching bands occur at 3262 cm^−1^; hydroxyl bending bands at 897 and 745 cm^−1^ and Fe-O stretching bands at 630 cm^−1^ are ascribed to goethite [50,51]. Shoulders at 1372 and 1324 cm^−1^ are assigned to calcium, zinc, and copper oxalates [51]. Figure 18c shows a secondary electron image of a sample excised from the remains of the glasses frame ETN073. The image shows a notable amount of cryptocrystalline particles deposited on the surface of the spectacles frame. The EDX analysis performed in point 1 provides a P/Ca molar ratio of 1.81 and a P/Zn molar ratio of 1.14. (Figure 18c). These P/Ca and P/Zn ratios suggest the presence of scholzite (CaZn_2_(PO_4_)_2_·2H_2_O).

### 2.5. Fungal Biodeterioration

**Figure 19:**
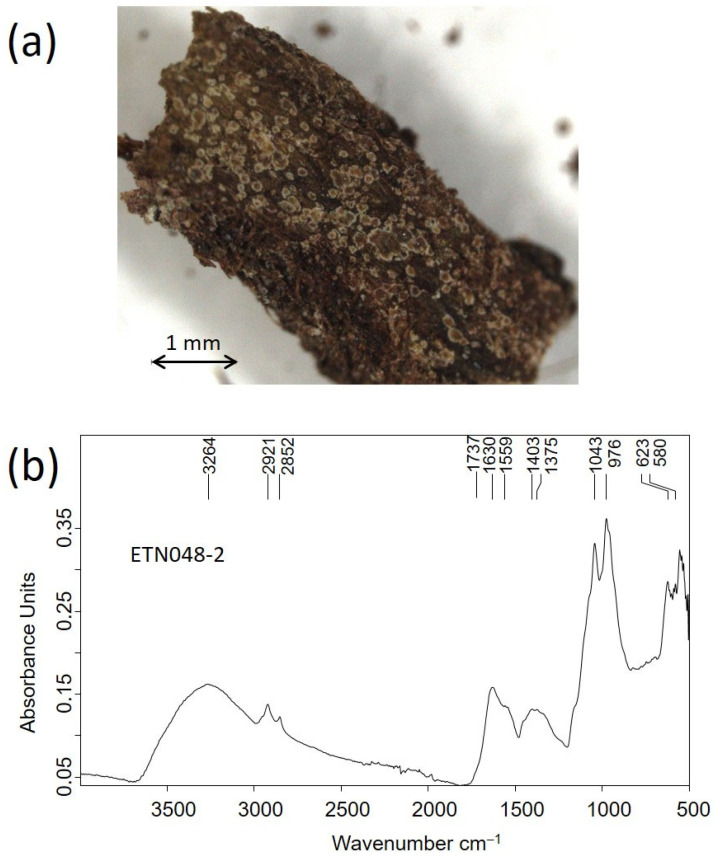
Knife ETN048: (**a**) microphotograph of the microorganisms formed in the surface of the sample of the knife; (**b**) IR absorption spectrum.

In the image shown in Figure 19a, spots of a few tenths of a millimeter of diameter of microbiological nature can be observed in a sample excised from the organic materials covering the tag knife. The IR spectrum acquired in this sample (Figure 19b) exhibits a band profile characteristic of fungi colonies with stretching and deformation bands of OH groups at 3264, stretching at methyl and methylene groups in lipids at 2921 cm^−1^ and 2852 cm^−1^, stretching vibrations at 1737 cm^−1^ ascribed at lipids, a band at 1630 cm^−1^ ascribed to amide I band in proteins, a band at 1559 cm^−1^ assigned to amide II band in proteins, deformation bands of C-H bounds at 1456^sh^, 1403, and 1375 cm^−1^, and an amide III band at 1321^sh^ cm^−1^ [52]. This last band and that other at 1630 cm^−1^ are also assigned to calcium oxalate’s symmetric and asymmetric stretching vibrations, respectively. Intense bands at 1043 cm^−1^ and 976 cm^−1^ are ascribed to polysaccharides. The latter and unassigned weak bands in the range 700–900 cm^−1^ (microorganism fingerprint) are characteristic of fungi [53].

### 2.6. Grave Soil

**Figure 20:**
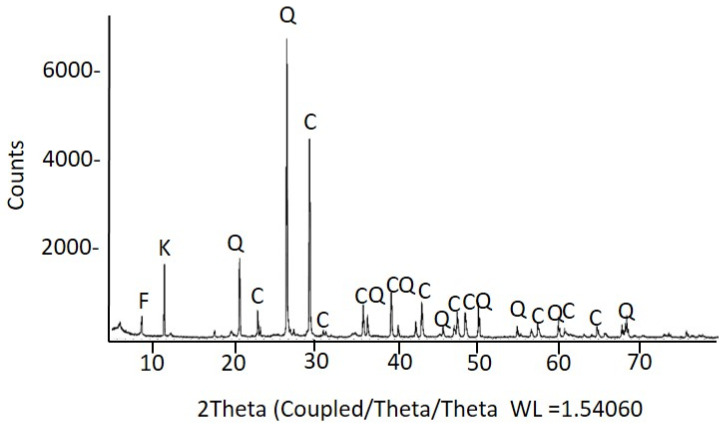
Diffractogram of a sample of the soil that filled the mass graves. C: calcite; Q: quartz; K: kaolinite; F: phlogopite.

In parallel to the personal items analysis, samples of the soil (SF2-01 to SF2-04) that filled the mass grave number 2 were analyzed using XRD (Figure 20), FTIR, and FESEM–EDX (Figure S6). The samples were taken in four points far from the corpse found in the saca placed at a depth of 4.75 m. The main compounds identified in the soil were calcite and quartz, accompanied by mica minerals, mainly of the phlogopite type, and kaolinite. The average elemental content of the soil, expressed as wt% and obtained from analysis in the area mode (see Materials and Methods section for details) using FESEM–EDX, was 41 ± 1 O %; 1.54 ± 0.03 Mg %; 6 ± 1 Al %; 19 ± 4 Si %; 0.3 ± 0.9 P %; 0.4 ± 0.4 S %; 1.7 ± 0.3 K %; 26 ± 5 Ca %; 0.4 ± 0.3 Ti %; 4 ± 1 Fe%. In addition, the spot analyses performed on individual grains enabled the identification of some of the most abundant minerals present in the soil from the experimental atomic percentages calculated using the ZAF method. Thus, potassium feldspars of experimental stoichiometry ((K,Mg)_1.00_(Al,Fe)_0.98_Si_3.26_O_8_), wollastonite (Ca_1.10_Si_1.00_O_4_), zircon (Zr_0.99_Si_1.00_O_4_), and gypsum (Ca_1.16_SO_4_.2H_2_O) could be identified. The presence of calcium apatite/hydroxyapatite and chloride could be noted by the corresponding emission lines of P and Ca. The apatite found in samples taken from the grave soil may be due to the proximity to human remains, which would have been the sources of phosphorus and calcium [54]. Still, it may also have a geological origin.

## 3. Discussion

### 3.1. Connecting the Materials Identified and the Manufacturing Technique

The primary purpose of this study was to identify the materials that composed the personal items collection as, in most cases, it was not possible to identify them using organoleptic methods due to the poor state in which they were found.

**Figure 21:**
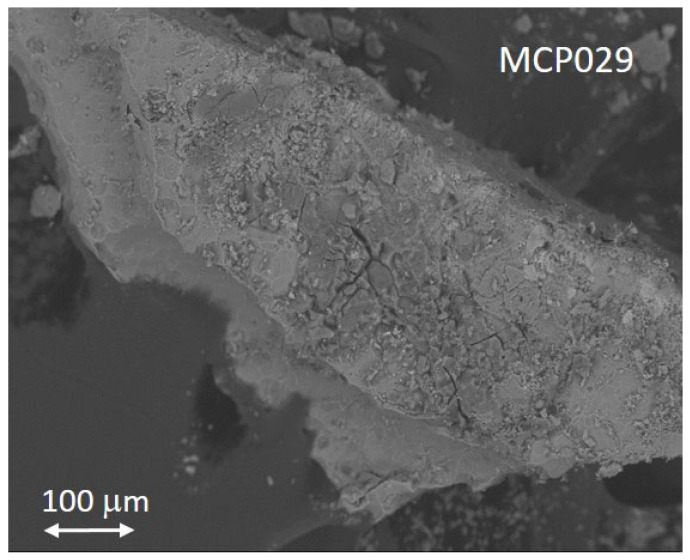
Secondary electron image of sample MCP029 corresponding to the aluminum devotional medallion.

The high level of oxygen detected in the sample MCP029 from the aluminum devotional medallion raises the question of whether this metal had a protective anodizing treatment. Anodizing is an oxidizing treatment of the metal surface that can be driven either electrochemically or chemically to form an aluminum oxide film much more stable and thicker than the aluminum oxide film formed naturally. The generated anodized oxide layer is thick, dense, and passive enough to avoid corrosion and abrasion of the metal surface. The Bengough–Stuart process, developed in 1923, was the first anodizing method consisting of a chromic acid–base treatment, and it was used to protect seaplane parts from corrosion [55]. That and other anodizing treatments became popular in the early 1960s [56,57]. The surface of anodized aluminum is characterized by two different morphologies depending on whether the formed oxide film is slightly soluble or completely insoluble in the electrolyte used. The former (i.e., sulfuric, phosphoric, chromic, and oxalic acids) produce porous films. In contrast, non-porous barrier films are obtained with the latter (i.e., neutral boric acid solution, ammonium borate, or tartrate aqueous solutions (pH 5–7), ammonium tetraborate in ethylene glycol, and organic acids such as citric, malic, or glycolic acids). The porous anodized surfaces have an outer nanoporous layer and an inner non-porous thinner layer that acts as a barrier in contact with the metal surface. Accelerated aging experiments simulating natural weathering have demonstrated that the corrosion of a non-treated aluminum surface is characterized by micro-cracks and pitting on the micrometer scale, whereas anodized surfaces exhibit a more diminished pattern in the nanometer scale [58]. Thus, this different behavior can provide clues about the manufacturing method used for making the devotional medallion MCP029. Figure 21 shows the secondary electron image of the sample MCP029 in which fractures measuring several tens of micrometers in length and depth are visible on the sample’s surface, indicating that this object was not subjected to an anodizing treatment. That would be consistent with the period in which it is supposed to have been manufactured, that is, the beginning of the 20th century until the beginning of the Spanish Civil War.

**Figure 22:**
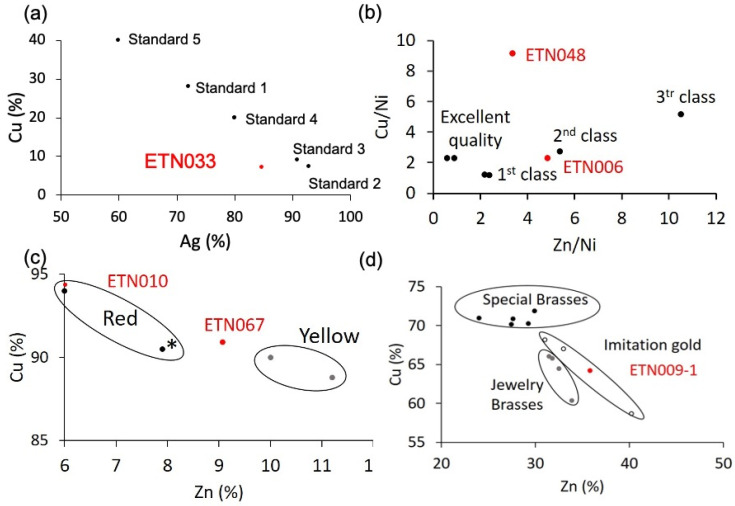
(**a**) Copper content versus silver content of standard Ag-Cu alloys and sample ETN033 excised of a silver ring; (**b**) Cu/Ni vs. Zn/Ni of standard alloys and samples ETN006 and ETN048 excised of a spoon and a knife; (**c**) Cu wt% vs. Zn wt% of standard jewelry brass alloys, and samples ETN010 and ETN067 excised from the pencil extender and the medal (* the alloy includes 1.6 wt% Pb); and (**d**) Cu wt% vs. Zn wt% of standard jewelry brass alloys, standard special brass alloys, imitation gold alloys, and sample ETN009-1 excised from the cigarette lighter. Black dots that correspond to experimental compositions corresponding to a specific reference alloy are framed with black continuous lines; red dots correspond to the experimental values found in the samples.

Copper alloys have prevailed among the metallic items. A silver copper alloy was used to make the ring ETN033. It is well known that the solubility of copper in silver is about 8–10 wt% at 780 °C (eutectic temperature) [59]. Nevertheless, when silver is alloyed with copper, only one proportion is known to result in a homogeneous melt, which is 72% silver and 28% copper (alloy standard 1). Proportions other than that give rise to segregation problems in the object. If the proportion of silver is higher than alloy standard 1, the central part of the object becomes enriched in silver, and the edges harden more quickly. On the contrary, if the silver proportion falls below 72%, the object’s core becomes Ag-poor, and the edges become Ag-rich [60]. For this reason, the alloys used in jewelry are around these values. According to Hiscox and Hopkins [60], the silver alloys usually used in jewelry are (Figure 22a) 77 parts Ag, 6 parts Cu (standard 2); 40 parts Ag, 4 parts Cu (standard 3); 4 parts Ag, 1 part Cu (standard 4), and, more specifically, alloys for chains are prepared with 3 parts Ag and 2 parts Cu (standard 5). The diagram depicting the Cu content vs. the Ag content in Figure 22a shows that the ring’s chemical composition is close to standards 2 and 3, with the highest silver contents. However, it is also observed that the ring has a lower silver content than these standard alloys. Suppose the composition of silver is compared separately, in that case, standard 4 is the closest to the experimental composition obtained for silver in the ring, but, in contrast, the copper content is notably lower than standard 4. This loss can be associated with leaching and reprecipitation processes in the burial soil. These processes are common in archaeological objects found in burials, and they have been widely studied and described. The formation of cuprite (Cu_2_O), tenorite (CuO), atacamite [CuCl_2_·3Cu(OH)_2_] chlorargyrite (AgCl), and stromeyerite [CuAgS] are among the corrosion products most frequently found [59,61,62,63,64,65]. Most of these researches have demonstrated that, in general, copper is more prone to undergo leaching. According to Ingo [65], the intimate contact between copper and silver grains in the Ag-Cu alloys, which have different electrochemical potentials, promotes couples strongly conducive to corrosion. In those microscopical systems, the copper phases become the anodic area, and preferential dissolution of copper occurs. These processes affecting selectively copper in Ag-Cu alloys could justify the discrepancies found in the experimental composition of the ring and the composition of the standard alloys, in particular, standard 4.

The samples ETN006 and ETN048 from the spoon and the knife were identified as copper ternary alloys composed of copper, zinc, and nickel. The proportion found among the three elements points towards German silver. This alloy is well known for being comparable to silver in terms of its visual appearance, improving its hardness and unalterability. Properties such as ductility, fusibility, or color vary according to the proportion in which the three metals of the alloy are combined. According to Hiscox and Hopkins [60], the usual composition of German silver consists of 50–66 parts of copper, 19–31 parts of zinc, and 13–18 parts of nickel despite the best composition for manufacturing spoons, forks, and knives consisting of 50 parts copper, 25 parts zinc, and 25 parts nickel. Other proportions that improve or reduce the alloy properties have also been used that incorporate small amounts of lead, iron, manganese, or tin. Figure 22b shows the diagram Cu/Ni vs. Zn/Ni in which the most common compositions of German silver are depicted according to the quality of the cutlery. The position of the sample ETN006 ascribes this alloy to the group of the second class. The sample ETN048 excised from the knife does not match any German silver alloy. This could be due to the sample’s poor quality and thick corrosion layer enriched in zinc and copper alteration products, which distort the result. This may lead to an overestimation of the copper content to the detriment of the actual zinc content, as shown by the position occupied by this sample in the diagram.

A ternary alloy Cu-Zn-Pb was identified in sample ETN010 excised from the pencil extender and sample ETN067 from the medal. A silver coating has also been identified in the pencil extender. The composition of both samples is depicted in Figure 22c, which shows the diagram of Cu % content vs. Zn % content. The diagram also shows the composition of common Cu-Zn and Cu-Zn-Pb alloys used in jewelry with characteristic red and yellow-reddish colors [60]. The chemical composition of sample ETN010 accurately matches that of one of the two red alloys, whereas the ETN067 sample exhibits an intermediate composition between red and yellow alloys. The lead content found in the samples would be explained by providing greater malleability during manufacturing. The pencil extender, also called pencil lengthener, is a small instrument used to facilitate the use of pencils when the small length hinders their use. The extenders are made out of metal or wood. Engineers, drafters, and artists currently used extenders to maximize the use of their favorite pencils. Due to its shape, the binary Cu-Zn composition was likely adapted by including a small amount of lead in the alloy, prioritizing malleability over other properties such as color to adapt it to the industrial process of manufacturing the body of the objects by molding or casting.

On the other hand, barite and a cobalt pigment were also identified in the medal, which is associated with the rest of the original pictorial decoration of the medal. Thus, it is possible that barite was used to decorate the white parts of the medal, and cobalt pigment was used to decorate the blue parts. Nevertheless, barite is a well-known pigment added as an extender by the paint manufacturers to provide opacity [66]. Identifying a blue cobalt pigment in the same sample as barite does not exclude that the latter was merely used as an extender of the cobalt blue pigment used for decorating in blue the medal.

The elemental composition found in the sample ETN009-1 excised from the metallic case of the cigarette lighter corresponds to a quaternary brass alloy Cu-Zn-Sn-Pb. In contrast, other internal parts of the lighter were made of steel (ETN009-2) (see Table 1). Brasses have been widely used in jewelry (costume jewelry) to make jewels at a much lower cost. Ordinary brass is more rigid than copper and better able to withstand stress. Annealed brass can be stamped and percussion processed. Concerning composition, copper and zinc are alloyed in brass at all proportions, with their properties varying depending on the temperature at which the alloy is produced. The alloys with the most significant resistance contain 20 to 30% Zn. If the concentration is raised to 60%, the resistance decreases, preventing mechanical uses. There are very ductile and malleable brasses, which, together with the different varieties of color, make them very suitable for artistic applications. The malleability varies with the composition of the two main elements, Cu and Zn, the temperature, and the addition of small quantities of certain metals such as lead and tin. Some alloys can be rolled at any temperature, and others can be used only when hot. Alloys with a 15 to 30% zinc content are suitable for drawing into wire. On the other hand, a series of alloys of copper and zinc have been developed that have been generically called gold imitations. These brasses have been widely used in jewelry (costume jewelry) to make jewelry at a much lower cost. These include chrysocolla, Mannheim gold, and mosaic gold. Figure 22d shows a diagram of Cu wt% vs. Zn wt% in which the copper and zinc content of a series of common brasses of use in jewelry and watchmaking (Stoberg, Hamilton, and watchmaking brass), special brasses (special French brass, French brass for molding and casting, English brass, Heegermuhl, and Ausburg brass), and gold imitations (chrysocolla, Manhein gold, and musive gold) in use in the first half of the 20th century are depicted [60]. The brass of the cigarette lighter ETN009-1 case has a high zinc content in the concentration range in which gold imitation alloys are also found. However, these alloys are usually ternary, including tin as a minor element in some cases and lead in others. Therefore, it is more plausible that the ETN009-1 alloy corresponds to a common quaternary brass such as Hamilton brass, which has a similar copper percentage (Hamilton brass, 64.5% Cu, 32.5% Zn, 0.3% Sn, and 2.7% Pb).

Silver was also detected in spot analyses from samples of the pencil extender (ETN010). Silver must have been applied as a coating on the brass body of the pencil extender to embellish it. At the beginning of the 20th century, the silver plating of brass objects was carried out using three different methods: dry, wet, and electroplating. The dry method consisted of rubbing the metal surface of the object with a paste that included silver salts (i.e., three parts silver chloride, three parts sodium chloride, two parts chalk, and six parts potash; or 1 part silver nitrate and three parts potassium cyanide; or three parts of silver chloride, eight parts of sodium tartrate, and 1 part of sodium chloride). The brass object was carefully cleaned with diluted nitric acid and ammonia and dried with sawdust. Finally, the silver layer was protected with lacquer. The resulting silver layer is not very durable, so this method only applies to objects that will not suffer much wear, such as barometer and thermometer scales. The wet method could be carried out following a cold or hot treatment. Cold treatment was carried out by immersing the object in a bath containing a liquid that, according to Boettegen’s formula, included two parts of silver hyposulfite, 1 part of ammonium chloride, and 20 parts of water. Another bath formula specific to brass consisted of 20 parts silver nitrate, 120 parts potassium cyanide, 30 parts washed gypsum, and 1000 parts water. A hot silver plating of brass was carried out at 80–90 °C [60]. The objects were immersed in enameled containers. The bath consisted of 10 parts of silver nitrate dissolved in 500 parts of water, which were mixed with 35 parts of potassium cyanide dissolved in 500 parts of water [60]. Until the end of the 1950s, the industrialization of electroplating would not begin in Spain since electrolytic coatings were only used in a very artisanal way in small manual installations, except in some state-owned companies of the National Institute of Industry (INI) such as SEAT automotive factory. The electroplating was carried out in electrolytic baths of silver and potassium cyanide. The anode was a silver bar of size proportional to that of the brass object that acted as a cathode [67,68]. The analysis of the ETN010 pencil extender has not provided any experimental evidence to determine which of these three methods was used to manufacture this object. However, the use for which it was intended required the application of a layer of wear-resistant plate, so it is very likely that the electroplating method carried out in artisan workshops was used. Still, the cold or hot wet plating method has also not been ruled out.

In the ring ETN042 and the button MCP001, calcium carbonate has been identified as the main component. Bivalve (Mollusca: Bivalvia) shells are composed of 95–99.9% calcium (CaCO_3_) and calcium–magnesium carbonates (CaMg(CO_3_)_2_) present in the form of calcite, aragonite, and dolomite in an organic matrix of conchiolin proteins with inorganic trace elements that vary according to the species and the surrounding environment [69]. Thus, the geogenic formation process in the first two and the biogenic in the last explains the identification of two different polymorphs of calcium carbonate, calcite, and aragonite, respectively. The IR absorption spectra obtained from the three samples can provide additional information beyond recognizing the constituent mineral species. This additional information relates to these materials’ degree of atomic order. It has been demonstrated that the atomic disorder in a calcium carbonate structure is closely related to the origin; that is, the process of calcium carbonate formation determines the final structure of the mineral: geogenic (limestone, chalk, and marble rocks), anthropogenic (mortar and plaster products of pyrotechnologies) or biogenic (eggshell, mollusk shells, coral, foraminifera). In mortars and plasters, lime is first produced by heating geogenic calcium carbonate to high temperatures. When the lime is applied in the construction, the lime is allowed to hydrate and absorb CO_2_ from the atmosphere. In the biogenic formation of calcium carbonate structures such as nacre, the bio(macro)molecules interplay with the precursor amorphous calcium carbonate (ACC) and drive the process toward the final crystal structures [70].

An indicator of the degree of the atomic disorder can be obtained by studying CO_3_ deformation vibrations ν_2_ and ν_4_ that exhibit the IR spectra acquired in calcium carbonate of different origins as the ν_4_ band is more influenced by the atomic disorder than the ν_2_ band. This can be quantified by measuring the intensity ratio *I*_2_/*I*_4_ of both ν_2_ and ν_4_ bands [71]. The higher the value of *I*_2_/*I*_4_, the greater the degree of disorder in the crystal structure. All this has given rise to an archaeometrical research line focused on distinguishing among calcites formed under diverse conditions based on the measuring of the *I*_2_/*I*_4_ ratio [72,73,74,75]. According to the literature [71,72,73,75], well-ordered calcite materials exhibit sharp and intense ν_2_ and ν_4_ bands. The *I*_2_/*I*_4_ ratio for well-ordered geogenic sparry calcite is around 2.0–2.2, and geogenic calcite ranges from 2.3 to 3.4. In anthropogenic calcite, the ν_4_ band maintains its shape without widening but diminishes the intensity to the point that in the amorphous calcite, the ν_4_ band does not appear [70]. The *I*_2_/*I*_4_ ratios found in anthropogenic mortars and plasters from lime formed at elevated temperatures (kiln at 700 °C or over) range widely between 3 and 7. Chu et al. [72] point out several causes: the original limestone may have been partially burned during the lime manufacture, and the resultant mortar is a mixture of original geogenic crystals and neoformed anthropogenic calcite grains. Limestone aggregates may have also been added to improve their binding properties. In addition, neoformed calcite in the mortar can dissolve and reprecipitate over time in the form of more ordered crystals. In biogenic materials, the ν_4_ band widens symmetrically around the band maximum more than the ν_2_ band. The *I*_2_/*I*_4_ ratios of biogenic calcite ranges from 2 to 3.6. In the biogenic formation of calcium carbonate materials, the short-range orders play a key role in crystalline polymorph selection. In mollusks, colloidal nanoseeds of immature ACC with random crystallographic orientations fuse by oriented attachment to form the mature tablet of aragonite [69].

The experimental *I*_2_/*I*_4_ values were 2.27 and 2.43 for the ETN053 ring and MCP001 biogenic nacre, respectively. In the two cases, the values found are in the low part of the experimental ranges found in the literature, which indicates that the calcite and aragonite are structurally well ordered at the atomic scale. This result suggests that the burial has not altered the nacre structurally. Therefore, the alterations caused must be associated with the interaction of the external environment. The organic matter coming from the decomposition of the deceased corpses has formed an ionomeric phase promoted by the soil moisture that impregnates the ring and has released the calcium ions of the surface and, finally, has formed carboxylate complexes [29]. In the button, the corrosion processes occur through interlaminar decohesion produced by the sorption of water present as soil moisture, which produces a significant loss in the mechanical resistance of the material.

Another interesting group of objects is formed by the two nit combs ETN060 and ETN058 and the spectacles frame ETN073. The IR spectra acquired in these samples indicate that a casein-formaldehyde (CF) resin was used to make these materials. CF is not consistently recognized in organoleptic examinations of heritage goods as it has a similar appearance to other types of semi-synthetic resins such as cellulose nitrate or acetate, hence, the importance of developing precise identification methods. A thorough examination of the IR spectra acquired on the samples reveals that the CF resins identified on all three objects were dyed. This result was confirmed by FESEM–EDX analysis (Figure S7 and Table S3). The dying with an earth pigment provided the tortoiseshell appearance to these personal items [66].

Until 1936, the range of plastic materials with practical applications was limited. However, using some of them as imitation materials of other more expensive materials from natural origin had already become widespread. An example is CF resin, which was used to make perfect imitations of semi-precious materials such as mother of pearl, tortoiseshell, ivory, horn, and ebony. Although the CF resin was synthesized in 1897 by the German printer Adolph Spitteler and his associate W. Krische, the milk plastic process was not patented until 1911 by the British company Syrolit Ltd. This plastic material became popular because, once manufactured, it could be ironed and cleaned wet or dry, making it able to manufacture buttons, knitting needles, fountain pens, and hair combs. The development of other petroleum-based polymers, such as polyvinyl chloride, polyethylene, polyester, acrylic, and melamine, in the first half of the 20th century, was progressively limiting the applications of this material [76]. This plastic material could be dyed with colorants during the processing of the object [77]. The coloring of CF plastic is carried out before the extrusion, although this process can be performed after manufacturing using many recipes. To dye the object after extrusion, the material, with the final shape already given, had to be wrapped in muslin and immersed in a bath of dye heated to 90 °C for at least 15 min [78]. The description of both procedures provides clues as to what procedure could have been used to stain the personal objects analyzed. Staining using a 90 °C hot bath requires water-soluble dyes, which would rule out the earth pigments and calcium carbonate extenders identified in the samples analyzed. In addition, these stains are superficial. This also seems to rule out this dyeing system for the objects analyzed.

Three natural products were identified in the personal item sharpener ETN012, rope handcuffs ETN045, and the contents of the little bottle LLUE001. Manufacture by the tin industry of manual pencil sharpeners began in the second half of the 19th century. At the same time, mass pencil production took place. Mechanical sharpeners were the subject of numerous patents registered worldwide between 1850 and 1900. At the beginning of the 20th century, various sharpeners were available according to needs, and their designs were perfected over time. The first portable pencil sharpeners were small, metal, and conical in shape adapted to the graphite pencil. Among the materials used in the first half of the 20th century for making sharpeners, metal, wood, and semi-artificial resins are included. Although there are no reports of pencil sharpeners made with CF or other semi-synthetic resin, this polymer was likely used to manufacture this object given the large number of applications it had at this time, some of which, such as combs or buttons, required, like the pencil sharpener, a specific mechanical and abrasion resistance. The analysis has resolved this item by demonstrating that, in this case, wood was chosen.

Esparto grass experienced two moments of splendor in the 20th century, coinciding with the two great world wars. The first, between 1914 and 1920, lasted until the 1930s. In both cases, the shortage and concomitant rise in the price of other fibers such as jute and hemp caused esparto grass to gain value. The rope handcuffs ETN045 found have the traditional characteristics of a simple three-strand braid. Each strand is made of five or six esparto fibers.

More interesting is the information provided by the item LLUE001. The name “Naranjil”, engraved on the glass bottle, is the trademark of a castor oil flavored with orange marketed by the company V. Llorens Sastre based in Valencia. Naranjil was a castor oil used as a purgative. It was registered in 1925 by Llorens and was on the market for most of the 20th century (Figure S8). Large pharmaceutical companies had not yet been widely settled in Spain then. It was common to find pharmaceutical products on the market for medicinal purposes based on natural products processed by doctors and pharmacists who patented and marketed them. Thus, a Naranjil competing trademark was the castor oil from the Camós Laboratory [79]. This product was produced in a traditional pharmacy in Algemesí (Valencia, Spain) that was run for decades by Mr. Fernando Camós Vidal, who died in 1986 [80].

### 3.2. Alteration Processes

The particular burial conditions have determined the main chemical and biochemical mechanisms that have altered the state of the materials comprising the personal items. Metallic objects have suffered electrochemical corrosion processes typical of burial environments in which the soil is cyclically saturated with humidity that subsequently decreases, giving way to a dry phase. In the humid phases, a thin layer of water is fixed to the surface of the metal, carrying salts and dissolved oxygen and acting as an electrolyte. The metallic ions released in this process reprecipitate on the object’s surface as salts with the available anionic species, carbonates, chlorides, sulfates, etc. Composition of the soil, pH, microstructure, and presence of cracks are factors that contribute to make complex the corrosion mechanisms.

**Figure 23:**
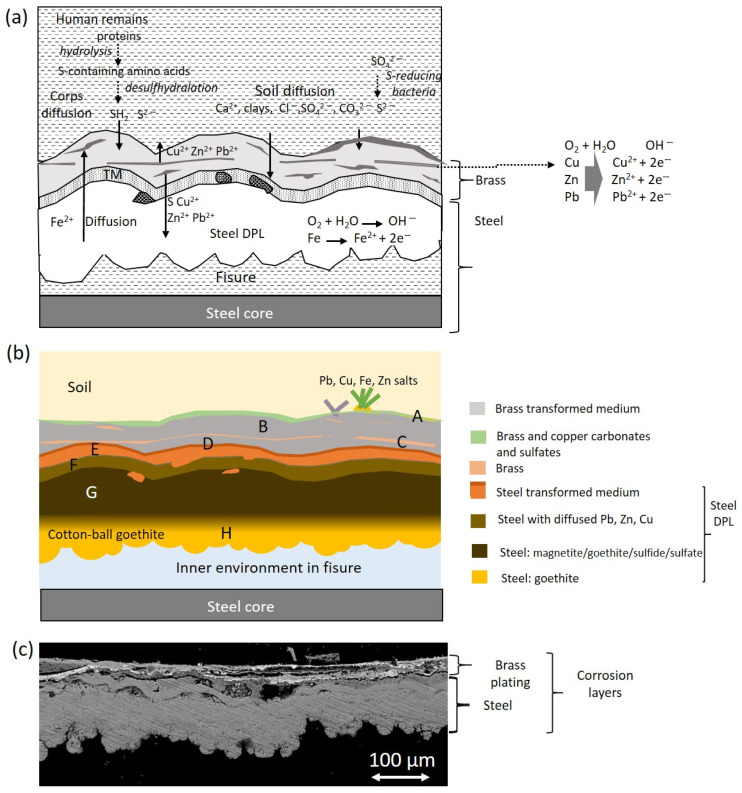
(**a**) Scheme of the processes that drive the corrosion and proposed redox and diffusion processes that have taken place in the case in the lighter ETN009; (**b**) regions with characteristic chemical composition identified by EDX and distribution of the brass and steel DPLs and TMs; (**c**) backscattered electron image of the studied sample that corresponds to the corrosion layers excised from the ETN009 case.

Figure 23a,c show a scheme of the electrochemical processes that have driven the steel and brass corrosion processes in the cigarette lighter ETN009. According to the literature [81], in iron and steel objects, the cathodic process is mainly undergone by oxygen that reacts with H^+^ ions to form water or with water to form hydroxyl ions, and the reduction of hydrogen is a minor process. On the other hand, the anodic process of the oxidation of iron to Fe^2+^ initially takes place in the surface of the unaltered metal in a separate point to the cathodic reaction. Once the process has started, the advance of the redox process depends on the environmental conditions. The pH of the medium, along with the O_2_ content, is an important factor that influences the behavior of the corrosion process. At a pH lower than six and the O_2_ level is high, the oxidation of Fe^2+^ ions to Fe^3+^ is slow, and most Fe^2+^ remains in solution. At low levels of O_2_ and pH values higher than six, the precipitated Fe(OH)_2_ is rapidly hydrolyzed to Fe(OH)_3_. Over time, the latter converts to goethite and can pass to magnetite [82]. With respect to the environment, the soil is a complex medium that contributes to influence the metal corrosion with different factors of geochemical type such as soil mineralogy, pH, electrical resistivity, and hydrological type such as water flow through ground layers. Thus, depending on the chemical and mineralogical composition of soil, the Fe^2+^ ions can precipitate and form Fe(OH)_2_ or can combine with exogenous ions from the soil such as Cl^−^, SO_4_^2−^, or CO_3_^2−^ yielding a corrosion layer with heterogeneous composition dominated by the more stable compounds [82]. Indeed, many analytical studies on the corrosion of archaeological iron artefacts agree that the main phases are goethite, magnetite, and maghemite (g-Fe_2_O_3_) and, less frequently, siderite and chloride (Fe_2(_OH)_3_Cl and akageneite (b-FeO(OH)). Neff et al. [83] establish that these materials form a compact and dense layer called the dense product layer (DPL), which forms from the unaltered metal and progressively gets thicker. Over the DPL is found the transition medium (TM) that contains both corrosion products and soil materials.

The multi-layered structure found in the ETN009 sample can be described according to the Neff’s model (Figure 23b). In the central part of the original steel foil of the case (G region), the Fe content is at ca 77%, maintaining a Fe/O molar ratio at ca 1. That indicates that the steel foil now is completely evolved into a steel DPL consisting of a mixture of oxides, hydrated oxides, oxyhydroxides, and carbonates that prevalently approximate the structure of magnetite and goethite in its outermost part. The small amounts of sulfur between 0.20 and 1.41 wt% suggests that the DPL also includes small amounts of iron sulfides and/or sulfates. Cotton-ball semicrystalline goethite prevails in the inner region L in contact with a great internal fissure [48].

A highly porous TM region (represented by areas D, E, and F) is recognized over the steel DPL, characterized by the presence of Cu, Zn, and Pb diffused from the upper brass layer and the progressive reduction of the Fe content until 59 wt%. That confirms the existence of decuprification, dezincification, and deleadification. The identification of S, Cl, Al, Si, K, Cu, and Zn together with Fe and O suggests that the steel TM has been formed by the cementing of the corrosion products and soil particles.

The outermost brass layer constitutes another singularity and adds complexity to this case study because two dissimilar metallic materials, brass and steel, are in electrical contact in the same electrochemical environment, and galvanic corrosion can occur. Here, Fe has a more negative electrochemical potential and becomes the anode, corroding faster than when steel is not in brass contact [41]. However, the results obtained in the brass indicate that this layer has also undergone intense corrosion processes that have produced various products. The O/Cu and S/Cu molar ratio values found in the remains of the original brass layer do not allow the type of compounds to be identified since they do not fit any particular composition. Despite this, it can be concluded that the material that forms this layer is probably a complex mixture with the prevalence of copper carbonates and oxides and some copper sulfate/sulfide.

The fact that copper sulfide/sulfate compounds were formed as alteration products in the mass graves is an interesting finding due to the rarity of these compounds in burial environments, and this is probably linked to the proximity of the personal items to human remains. That raises two questions: first, the source of this element, and second, the mechanism by which Cu-S compounds are formed. Regarding the source of S, a possible origin would be the dissolution of gypsum in the soil, which has been confirmed by analyzing several samples taken from different points in the mass graves. These sulfates could combine directly with the copper ions extracted from the brass layer by ion-exchange processes to form a copper sulfate such as brochantite. A second pathway forms sulfides from the soil sulfates through dissimilatory reducing microorganisms, i.e., sulfate-reducing bacteria [84,85,86]. This second mechanism is frequent in iron objects found in waterlogged archaeological sites and results in the formation of pyrite (FeS_2_) [84,87]. The iron reduction is coupled with organic matter or S^2−^ oxidation. Similarly, the human remains in the mass grave could provide a microenvironment for the artifacts close to them. The easy oxidation undergone by the organic matter of the soft parts mediated by microorganisms could promote the reduction of SO_4_^2−^ to S^2−^. Then, the S^2−^ could combine with copper ions, resulting in the unstable covellite (CuS) that further formed chalcanthite (CuS_2_) and subsequently was oxidized to the most stable copper sulfate brochantite (Cu_4_SO_4_(OH)_6_) in the presence of soil moisture [88]. This mechanism has been proposed by Muro et al. [89], who identify brochantite in copper objects found in a burial tumulus containing human remains. An alternative source of S^2−^, tentatively proposed here, could be the H_2_S release due to the decomposition of the proteins from the human remains close to the artifacts. Some proteins include amino acids such as cysteine and cystine that contain sulfur atoms. During the decomposition of the protein, these amino acids undergo a desulfhydration process that reduce them to form ammonia, thiols, pyruvic acid, hydrogen sulfide, and sulfides. Hydrogen sulfide release is favored in anaerobic environments such as grave soils [90]. If the environmental conditions in the grave turn aerobic over time, the transformation of sulfide in sulfate occurs [91]. On the other hand, it is well known from many studies of the corrosion mechanisms of copper, bronze, and other copper alloys exposed to the polluted air that exposition to hydrogen sulfide forms copper sulfides and specifically forms brochantite with high levels of humidity up to 70% RH [88].

The morphology exhibited by the brass shown in Figure 16 and Figure 23c suggests that the original brass layer would have become disjointed and fragmented due to local differences between the development of the corrosion process. The in situ formation of corrosion products combined with the diffusion processes of exogenous and endogenous species would have contributed to the micro-locally differentiated corrosion processes and rates and the dislocation of the brass layer. The original brass layer now is distorted and discontinuous (areas C) and completely converted to corrosion products characterized by high levels of Cu and O and the presence of S at a lesser extent (averaged molar ratios O/Cu = 2.9 and S/Cu = 0.6). A TM (areas A and B) associated with the brass wraps the remains of the original brass layer and is characterized by a significant Cu content, the presence of Zn and Pb diffused from the brass layer, Fe diffused from the case, and soil tracer elements such as Cl, S, Al, and Si coming from the soil.

The cryptocrystalline particles deposited in the sample ETN073 from the spectacles frame (Figure 18c) identified as scholzite would have been formed from the Zn^2+^ ions diffused from the metal glasses frame as result of dezincification in parallel to the calcium hydroxyapatite dissolution from the bones of the human remains disposed close to the spectacles. Similar processes have been reported in a burial tumulus where the deceased remains resulted in the formation of libethenite (Cu_2_(PO_4_)(OH)) and sampleite (NaCaCu_5_(PO_4_)4Cl.5H_2_O) [92].

The presence of calcium and copper oxalates, observed in some objects such as the spoon ETN006 and the knife ETN053, is associated with the action of microorganisms that have colonized these objects. It is possible that the presence of the remains of organic matter due to the alimentary use of these objects, which during burial could have been an ideal nutrient for certain fungi, has favored the development of this alteration mechanism, not observed in the rest of the objects.

However, without a doubt, the mechanism of alteration that has stood out in this set of objects has been the presence of adipoceres. It is worth highlighting the identification of two main types of adipoceres with a differentiated chemical composition: the nit comb ETN058 is a representative example of acidic adipoceres characterized by the predominance of fatty acids. The cigarette paper in a matchbox F111-141 and the rope handcuffs ETN045 are examples of the second type of adipoceres characterized by the majority presence of calcium salts of fatty monoacids. The fatty acids in contact with the surface of brass belongings have formed, at lesser extent, more specific adipoceres where zinc carboxylic complexes are prevalent. These last adipoceres have contributed to the corrosion of brass objects. The IR spectrum ETN073-2 acquired in a sample of adipoceres from the brass spectacles frame provides evidence of such a corrosion mechanism. Also noteworthy is the predominance of the monoacid acid and monoacid salt type compared to the diacid type, which is only found associated with Zn salts formed from brass. The lower presence of zinc salts compared to calcium salts is due to the more ubiquitous nature of calcium ions in a calcite-rich soil. Another aspect worth noting is the absence of copper or iron salts, which might be expected given the copper- and iron-rich composition of many of the personal items and which contrasts with the unexpected identification of zinc salts if it is considered that the latter is a minor component of the brass alloys. This fact is explained by the ease with which brasses with high Zn content of over 20% undergo dezincification when in contact with acidic or alkaline solutions. Among the factors that could promote dezincification in the brass personal belongings found in the mass graves can be the formation of earth deposits on the brass surface or at crevices, allowing differential aeration cells to form mildly aggressive aqueous soil mediums with a high chloride content or a high or low pH [93,94]. In the case of the zinc salts found in contact with the eyeglass frame, it is evident that this process involved a prior dezincification stage, which could have been facilitated by the presence of moisture in the soil and quicklime incorporated after depositing each saca, which would significantly raise the pH of the medium, favoring the corrosion of the brass by dezincification.

The extent of the adipoceres infiltrating the items and the possibility, as just described, of prolonging irreversible alteration processes in both organic and metallic materials required the adoption of specific intervention treatments. Sanitization was the first step in the conservation process. The larger superficial deposits of adipoceres that detached easily from the item were mechanically removed on a case-by-case basis. However, many objects were infiltrated of adipoceres to different extents, especially those made of organic materials. In such instances where mechanical removal was not feasible, a natural and fully sustainable product, melaleuca oil (tea tree essential oil), was used. This product, when prepared as a hydroalcoholic solution (70% ethanol and 30% water), can act as a biocide due to its fungicidal and bactericidal properties [95].

## 4. Materials and Methods

### 4.1. Optical Microscope

Stereoscopic microscope Leica GZ6 (X10-X50) and Leica DM2500 P (Leica Microsystems, Heidelberg, Germany) were used for preparing and examining cross-section samples, respectively. IC80HD Leica Digital FireWire Camera (DFC) with Leica Application Suite (LAS) software version 3.3.0 has been used to acquire and process digital images.

### 4.2. FESEM–EDX

A FESEM Zeiss (Orsay Physics, Fuveau, France; Kleindiek, Reutlingen, Germany; Oxford Instruments, Abingdon, UK) model Auriga compact equipment was used for the FESEM–EDX examination. The X-ray microanalysis was performed using an Oxford-X Max X-ray microanalysis system coupled to the FESEM. Operation conditions in the FESEM–EDX are as follows: a voltage of 20 kV, a current beam of 3.76–3.83 μA, and resolution of 127 eV at 5.9 keV. X-ray detector operates at a working distance of 6–7 mm. The electron beam was always disposed perpendicularly to the polished surface of the cross-sections. The same samples examined under an optical microscope were mounted on aluminum disks and carbon-coated before acquiring images and X-ray spectra. EDX analysis was performed with the Aztec software version 3.3sp1. Quantitative compositions were made from X-ray spectra acquired in point-mode acquisition. The wt% values of the different elements were calculated by applying ZAF method of correction of interelemental effects on intensity values. Three X-ray spectra from the selected area, of a few hundred of µm square, were acquired for quantitative analyses. The stoichiometry of the mineral was deduced from the molar ratio among the identified elements, which is provided by the quantitative processing of the detected X-ray counts. Other details concerning operation conditions are provided in Table S4 [96,97,98].

### 4.3. FTIR Spectroscopy

The mineral and molecular composition of the personal items was characterized by Fourier Transform Infrared Spectroscopy (FTIR) in attenuated total reflectance (ATR) with a Vertex70 spectrometer (Bruker Optik GmbH 2012, Ettlingen, Germany) with MKII Golden Gate with an ATR accessory (Specac^®^, Orpington, UK). This instrument is equipped with an FR-DTGS (fast recovery deuterated triglycine sulfate) temperature-stabilized coated detector and an MKII Golden Gate Attenuated Total Reflectance (ATR) accessory (Specac Ltd., Orpington, UK). The IR spectra were acquired by collecting 32 scans at a resolution of 4 cm^−1^. The spectral range scanned was 500–4000 cm^−1^. The spectra were processed using the OPUS software version 7.0 (Bruker Optik GmbH 2012, Ettlingen, Germany). The samples were directly analyzed or, if necessary, powdered in a small agate mortar, and a small amount of the powdered mixture was deposited on the diamond window.

### 4.4. XRD

Diffractometer Bruker model D8 Advanced A25 equipped with a Lynxeye fast detector and an X-ray tube that emits Cu Ka radiation. The operating conditions for the X-ray emission tube were 40 kV and 40 mA, and the acquisition conditions for the patterns were 5–80° 2q with an exposition time of 0.8 s.

### 4.5. Preparation of Samples

FESEM–EDX analyses were performed from the samples directly mounted on aluminum disks and C coated. The cross-section of the sample ETN009 was prepared by embedding in polyester resin (Glasspol S.L, Valencia, Spain) and polished with SiC foils with grid sizes of 220, 500, 2400, and 4000 (Struers, Germany) in a Struers LaboPol-20 instrument. FTIR spectroscopic analyses were performed on samples powdered in an agate mortar.

### 4.6. Ethical Considerations and Procedures

This study is based on exhumed objects from the research works carried out in the Municipal Cemetery of Paterna by the ArqueoAntro Scientific Association, a team dedicated to Management Archaeology, Historical Memory, Missing Children Cases, Training Courses, and Forensic Expertise. This association is hereby established under Spanish Organic Law 1/2002, of 22 March, and complementary regulations, with legal personality and full capacity to act, and with no profit motive. All exhumations were developed following the forensics protocols and legal authorizations and with the consent and participation of the family associations of victims [11]. Additionally, the project from which this study derives involved an interdisciplinary curatorial team comprising experts in exhumation processes, heritage management, public memory policies, and museum management [9]. Most importantly, the victims’ families were part of this project, which allowed the technical team to work, analyze, and publish our research on the exhumed objects. Regarding ethical responsibilities involved in handling, analyzing, and publishing data from mass graves and given the sensitive nature of the materials [15], the studio was implemented with maximum respect for the victims and their living relatives. Furthermore, the technical team established a close relationship with the families and relatives, who followed on-site year after year the exhumation works so that all the operations and scientific and technical procedures had their approval [23].

## 5. Conclusions

This research opens a new scenario in which multi-technique approaches based on the combination of fast and quasi-non-invasive techniques such as optical microscopy, FESEM–EDX, XRD, and FTIR spectroscopy are a suitable alternative to characterize materials and alteration processes in instances in which the analysis of a large number of archaeological objects with a diversity of materials of both organic and inorganic nature and alterations is demanded. Thus, inorganic materials such as nacre, limestone, pigments, and different brasses and silver plating have been identified. The organic materials identified are wood, esparto, cotton yarns, castor oil, and casein formaldehyde. The study has also provided data on how these objects were manufactured. In addition, typical metal corrosion processes have been identified with other unusual ones, such as the formation of brochantite and scholzite due to the proximity to the deceased corpses in decomposition. The generation of adipoceres during the decaying of the corpses has resulted in the formation of metal–carboxylate complexes and, mediated by microorganisms, metal oxalates, prevalently with calcium ions. Therefore, the analyses performed have provided the restorers with information on how to apply the most appropriate intervention treatments.

The study also provided clues of the forensic type, which are of interest to historians in finding out what life was like for the owners when they were imprisoned. The carefully carved stone rings reveal how the prisoners spent their long hours of imprisonment. Some of them entertained themselves with manual work that was not devoid of artistic value. The pencil extender and sharpener found provide similar information, suggesting that they wrote down thoughts and ideas, simply wrote letters to inform their families about their situation, or wrote farewell letters in the knowledge of their fateful end. Other items have more mournful implications. Unlike the previous items, it is unlikely that a prisoner would have been allowed to have a rope made of esparto grass, confirming as has been documented that it was used as handcuffs to immobilize the prisoner during transfer and execution. The small “Naranjil” bottle could have been used for medicinal purposes. However, it could also have been used to insert a piece of paper with the name of the prisoner to allow them to be identified in the mass grave once the execution had taken place. That made it easier for the family to make a subsequent claim. That seems to have been a common practice among the prisoners at that time, given the practices of the Francoist army [99,100], and this research has contributed to confirming such a hypothesis.

## Figures and Tables

**Table 1:** Elemental composition (weight %) of the metallic items analyzed.

Ref.	Elemental Composition (wt%)							
	Cu ^a^	Zn	Ni	Pb	Sn	Fe ^a^	Al ^a^	Ag ^a^
ETN006	50.5 ± 3	19.1 ± 3	8.3 ± 0.5	n.d.	n.d.	n.d.	n.d.	n.d.
ETN009-1	62.7 ± 0.9	37.3 ± 0.9	n.d.	5 ± 1 ^b^	15.9 ± 0.5 ^b^	n.d.	n.d.	n.d.
ETN009-2	n.d.	n.d.	n.d.	n.d.	n.d.	72.1 ± 0.7	n.d.	n.d.
ETN010-1	71.7 ± 0.5	4.6 ± 0.5	n.d.	1.51 ^b^	n.d.	n.d.	n.d.	4.5 ^b^
ETN010-2	n.d.	n.d.	n.d.	n.d.	n.d.	77.7 ± 0.9	n.d.	n.d.
ETN033-1	7.2 ± 0.4	n.d.	n.d.	n.d.	n.d.	n.d.	n.d.	84.7 ± 0.5
ETN033-2	6.6 ± 0.5	n.d.	n.d.	n.d.	n.d.	2.7 ± 0.3	n.d.	28.3 ± 0.6
ETN048	53.6 ± 0.9	11.8 ± 0.9	7.9 ± 0.6	n.d.	n.d.	n.d.	n.d.	n.d.
ETN067	90.9 ± 0.7	9.1 ± 0.4	n.d.	1.6 ^b^	n.d.	n.d.	n.d.	n.d.
MCP029	n.d.	n.d.	n.d.	n.d.	n.d.	n.d.	52.9 ± 0.2 ^a^	n.d.

n.d.: not detected; ^a^ oxygen wt% complete to 100% the sample elemental composition; ^b^ value corresponding to a spot analysis.

**Table 2:** IR bands identified in the sample ETN053 and assignment to chemical groups and compounds [28,29,30].

Assignment and IR Band Position (cm^−1^)	Material Identified
2ν_2_ + ν_4_ CO_3_^2−^ stretching (2509), ν_1_ + ν_4_ CO_3_^2−^ stretching (1797), ν_3_ CO_3_^2−^ asymmetric stretching (1398), ν_2_ CO_3_^2−^ asymmetric deformation (874), ν_4_ CO_3_^2−^ symmetric deformation (712)	Limestone
ν Si-O symmetric stretching (1113^sh^), ν Si-O-Si antisymmetric in-plane stretching 1027), δ (Al_2_OH) bending (915, 937), Si-O vibrations (748^sh^, 694)	Dioctahedral 1:1 clay
ν Si-O-Si stretching (1155^sh^, 1086^sh^), Si-O symmetric stretching (799, 777), Si-O of SiO_4_ symmetric bending (694)	Quartz
ν CH_2_, CH_3_ asymmetric and symmetric stretching (2918, 2851), ν C=O stretching (1730); calcium carboxylate stretching (1574)	Lipid matter

## Data Availability

The data presented in this study are available on request from the corresponding author.

## Supplementary Materials

The following supporting information can be downloaded at https://www.mdpi.com/article/10.3390/molecules30132783/s1. Figure S1: IR absorption spectrum acquired in sample ETN048 corresponding to a binding substance used for fixing the cotton yarns to the knife tag; Figure S2: IR absorption spectrum acquired in sample ETN060 corresponding to a nit comb; Figure S3: IR absorption spectrum acquired in sample ETN073 corresponding to the spectacles frame; Figure S4: IR absorption spectrum acquired in sample ETN045-2 excised from a rope; Figure S5: IR absorption spectrum acquired in sample ETN073-2 excised from spectacles frame; Figure S6: X-ray spectrum obtained in the sample SF2-01 from the soil of the mass grave number 2; Figure S7: X-ray spectrum obtained in the sample ETN060 excised from a nit comb. The emission line profile corresponds to an earth pigment; Figure S8: Label of the bottles LLUE001; Table S1: Description of the analyzed belongings; Table S2: Elemental composition (wt%) found in the different layers of the cross-section of sample ETN009; Table S3: Chemical composition corresponding to ETN060 sample excised from the nit comb. st: wt% sigma; Table S4: Experimental working conditions of FESEM–EDX.

## Abbreviations

The following abbreviations are used in this manuscript:

l’ETNO	Museums of Ethnography
GC–MS	Gas chromatography–mass spectrometry
OM	Optical microscopy
SEM	Scanning electron microscopy
FESEM–EDX	Field emission X-ray microanalysis scanning electron microscopy
FTIR	Fourier transform infrared spectroscopy
XRD	X-ray diffraction
C8:0	Octanodioic acid
C9:0	Nonanodioic acid
C14:0	Tetradecanoic acid
C16:0	Hexadecanoic acid
C18:0	Octadecanoic acid
C20:0	Eicosanoic acid
ACC	Amorphous calcium carbonate
ZAF	Z—stopping power, backscattering factor and X-ray production power (Z), absorption (A) and fluorescence (F)
CF	Casein formaldehyde
DPL	Dense product layer
TM	Transition medium
ATR	Attenuated total reflectance
FR-DTGS	Fast recovery deuterated triglycine sulfate
