# Supplementary material for: Between Heritage Conservation and Forensic Science: An Analytical Study of Personal Items Found in Mass Graves of the Francoism (1939–1956) (Spain)
Source: Molecules. 2025 Jun 27;30(13):2783. doi: 10.3390/molecules30132783 (PMC12251485; doi:10.3390/molecules30132783)
Supplement: Supplementary file 1 [file molecules-30-02783-s001.zip › molecules-3647392-supplementary.pdf]

Supplementary electronic material

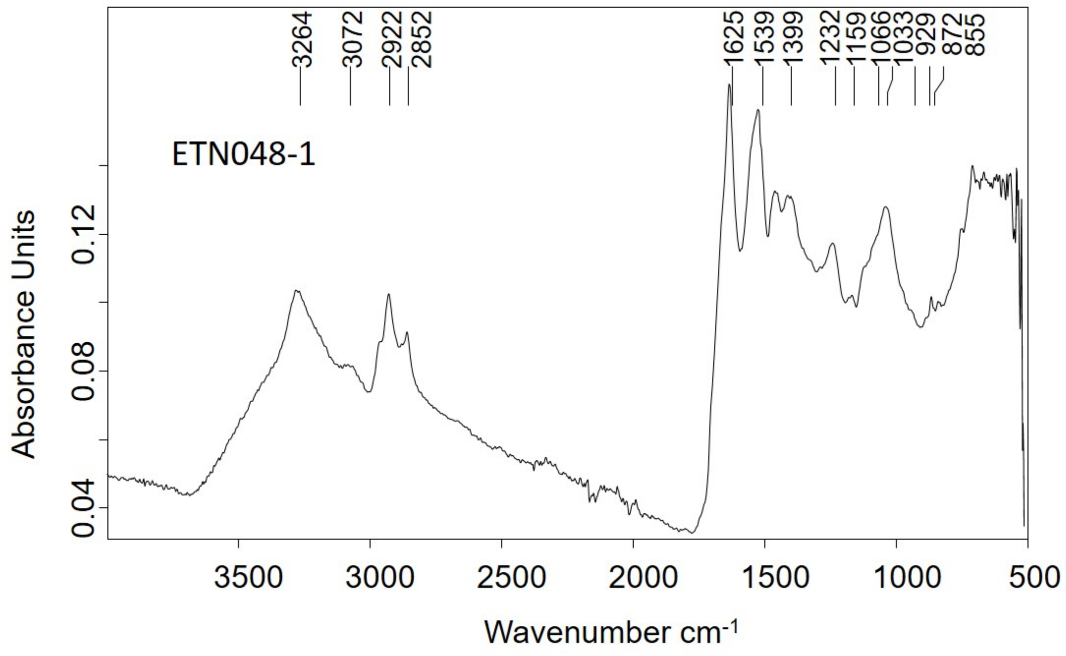

Figure S1. IR absorption spectrum acquired in sample ETN048 corresponding to a binding substance used for fixing the cotton yarns to the knife tag.

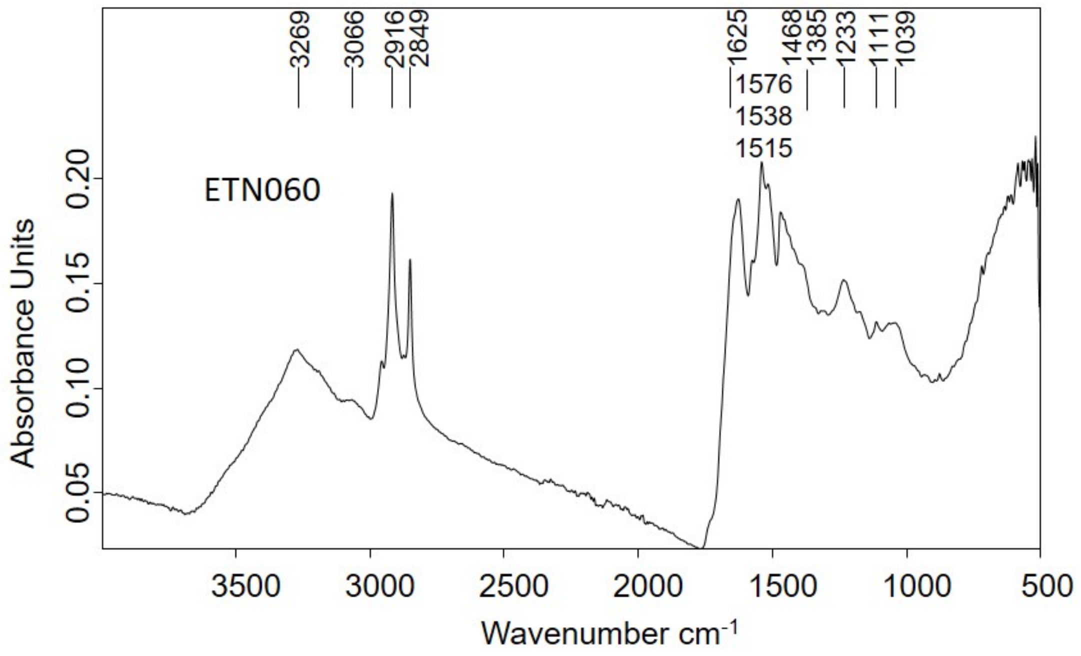

Figure S2. IR absorption spectrum acquired in sample ETN060 corresponding to a nit comb.

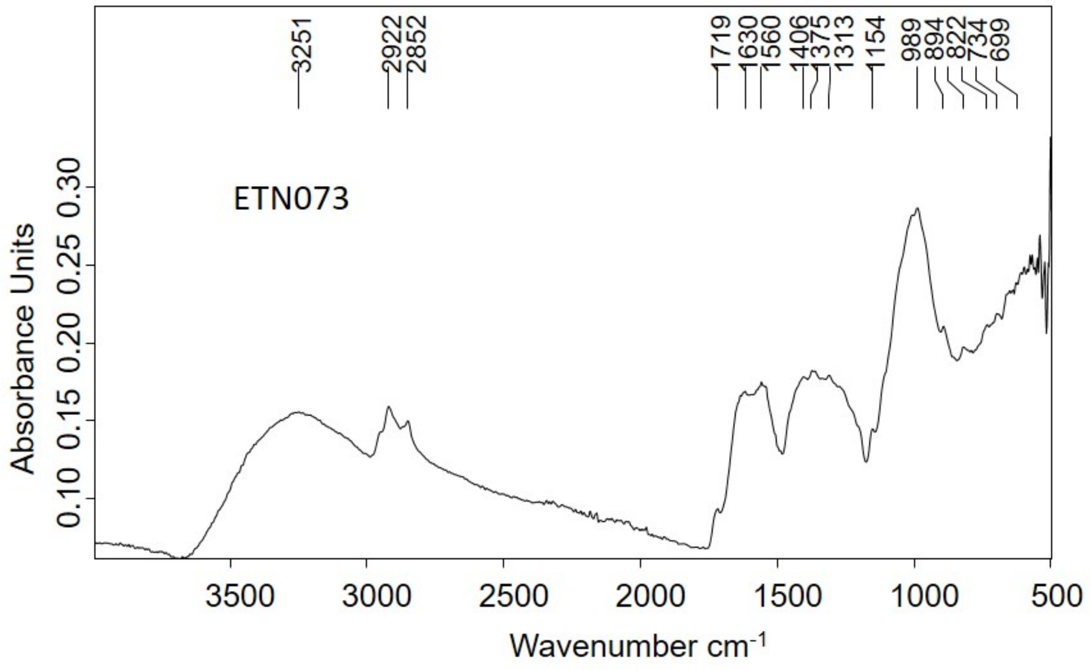

Figure S3. IR absorption spectrum acquired in sample ETN073 corresponding to the spectacles frame.

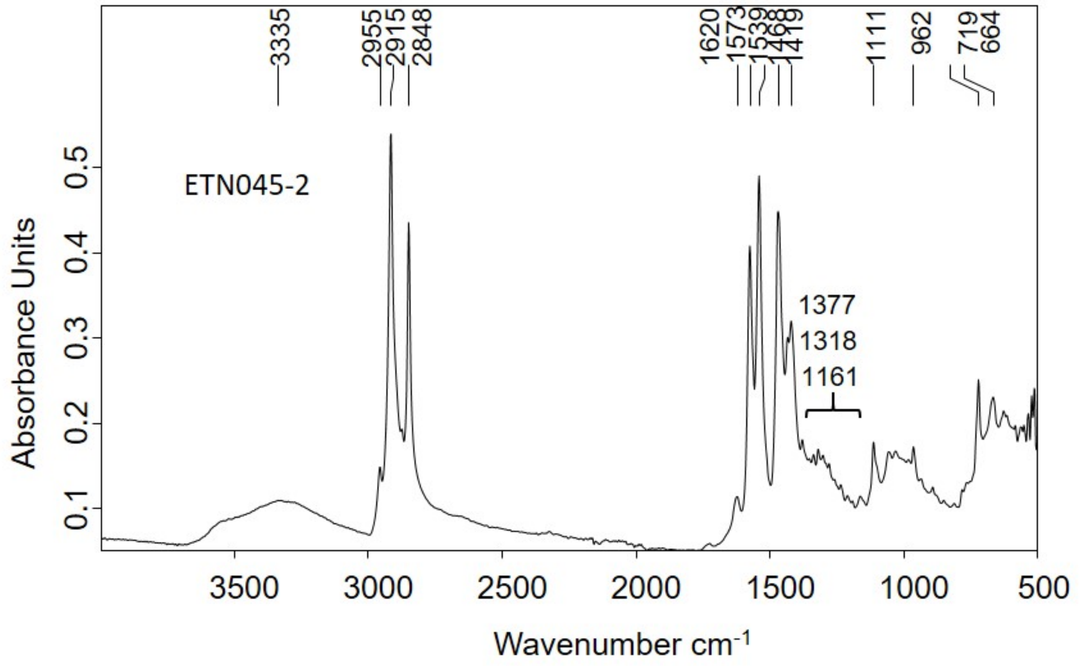

Figure S4. IR absorption spectrum acquired in sample ETN045-2 excised from a handcuffs rope.

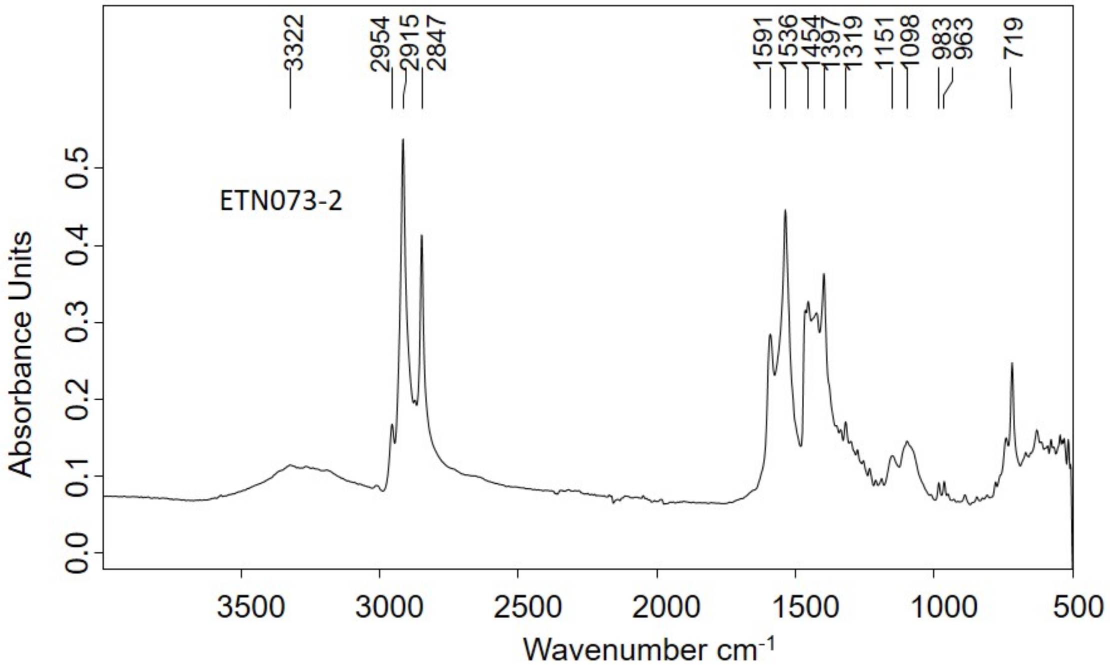

Figure S5. IR absorption spectrum acquired in sample ETN073-2 excised from spectacle frames.

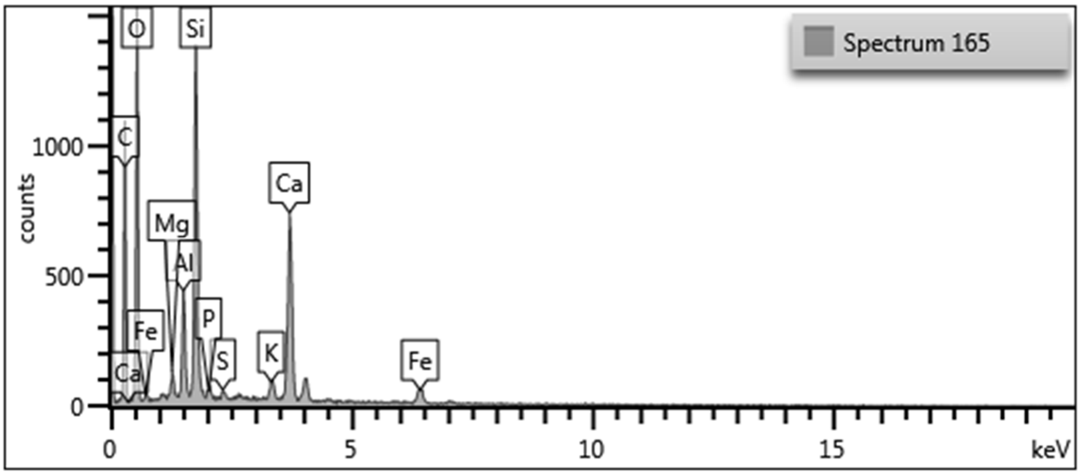

Figure S6. X-ray spectrum obtained in the sample SF2-01 excised from the soil of the mass grave number 2.

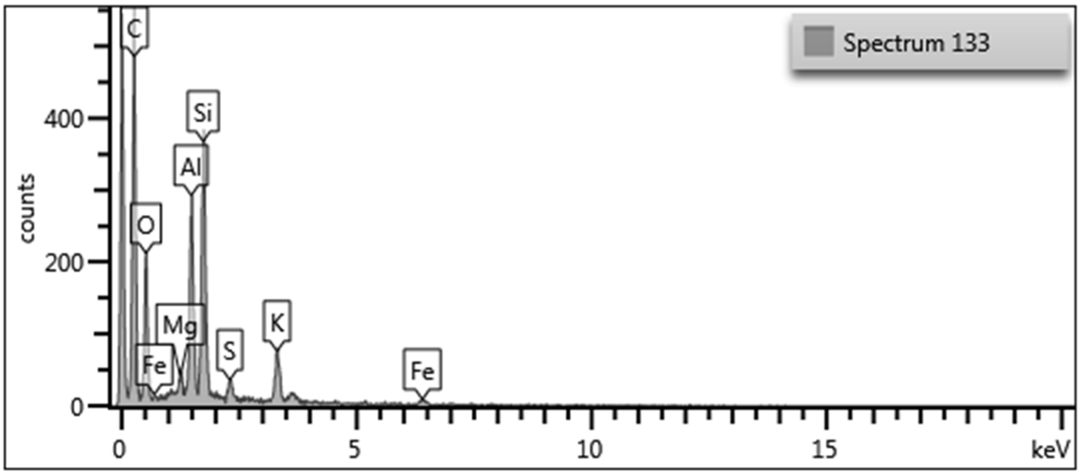

Figure S7. X-ray spectrum obtained in the sample ETN060 excised from a nit comb. The emission line profile corresponds to an earth pigment.

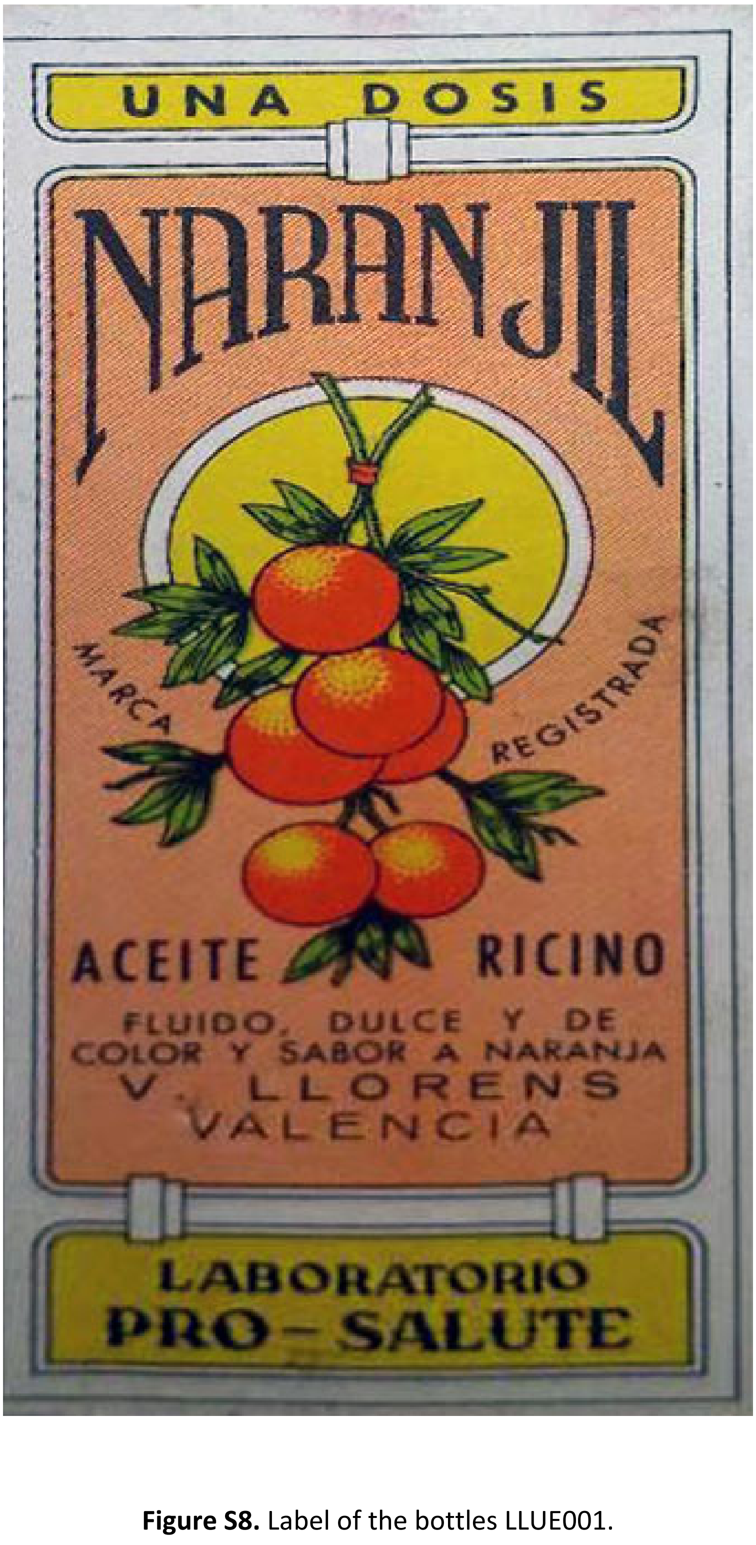

Figure S8. Label of the bottles LLUE001.

Table S1. Description of the analyzed belongings.

Ref	Description	Deceased	Mass grave	Objectives-techniques**					Observations
				Materials	Alterations	Manufacture	Function	Provenance	
ETN006	Spoon	43	115	FE	OM, FE, IR	FE	FE	FE	
ETN009	Cigarette lighter	131	115	FE	OM, FE, IR	FE	FE	FE	
ETN010	Pencil extender	144	115	FE	OM, FE, IR	FE	FE	FE	
ETN012	Pencil sharpener	107	115	IR	OM, IR	OM, IR	OM, IR	OM, IR	
ETN033	Metal ring	73	112	FE	OM, FE, IR	FE	FE	FE	
ETN045	Rope	92 Mr. José Peiró i Grau	112	IR, FE	OM, FE, IR	OM, IR	OM, IR	OM, IR	
ETN048	Knife	15	100	FE	OM, FE, IR	FE	FE	FE	
ETN053	Stone ring	28	94	FE, IR	OM, FE, IR	OM, FE,	OM, FE,	OM, FE,	
ETN058	Nit comb	95	112	IR, FE	OM, FE, IR	IR, FE	IR, FE	IR, FE	
ETN060	Nit comb	66, Mr. Juan Bautista Solanes i González	112	IR, FE	OM, FE, IR	IR, FE	IR, FE	IR, FE	
ETN067	Medal	68	128	FE	OM, FE, IR	FE	FE	FE	
ETN073	Spectacle frames	83	111	FE, IR	OM, FE, IR	FE, IR	FE, IR	FE, IR	
F111-141	Cigarette paper	141 Mr. Vicente Orti	111	IR	OM, IR	OM, IR	OM, IR	OM, IR	Contained in a matchbox with matches
LLUE001	Organic remains	14, Manuel Lluesma I Masiá	645	IR	OM, IR	OM, IR	OM, IR	OM, IR	Found inside a glass bottle with the inscription "*Naranjil Purgante ideal*"
MCP001	Button	19	114	OM, IR, FE	OM, IR, FE	OM, IR,	OM, IR	OM, IR	
MCP029	Devotional medallion	176	114	FE	OM, FE, IR	FE	FE	FE	Rests of a rope drawstring and textile fibers from clothes adhered to the medallion
MCP030	Ring	73	112	FE	OM, FE, IR				
SF2-01	Soil		2	FE, D	OM, FE, IR				4.75 m depth far from corps
SF2-02	Soil		2	FE, D	OM, FE, IR				4.75 m depth far from corps
SF2-03	Soil		2	FE, D	OM, FE, IR				4.75 m depth far from corps
SF2-04	Soil		2	FE, D	OM, FE, IR				4.75 m depth far from corps

*Number of the cemetery niche; ** OM: optical microscopy; FE: FESEM-EDX; IR: FTIR spectroscopy; D: XRD.

Table S2. Elemental composition (wt %) found in the different layers of the cross-section of sample ETN009.

	Content (wt %)							
Element	A	B	C	D	E	F	G	H
O	21.9	28.6	34.6	25.2	22.6	23.0	22.5	22.6
Al	0.69			2.05				
Si	1.27	0.85		2.73				
S	1.31	7.33	14.5	0.89	0.93	1.13	0.2	0.39
Cl	4.04							
Fe	4.8	20.1	2.53	59.0	68.0	69.0	77.3	77.0
Cu	65.9	43.2	48.4	4.4	3.69	1.85		
Zn				3.32	3.07	5.77		
Pb				2.32	1.57			
	Distance to the surface (μm)							
	4	15	29	30	40	50	70	120

Table S3. Chemical composition corresponding to ETN067 sample excised from the nit comb. st: wt % sigma.

**Element**	**wt%**	**st**
O	46.69	
Mg	1.78	0.29
Al	14.89	0.53
Si	22.95	0.65
S	2.39	0.32
K	8.26	0.49
Fe	3.05	0.69
Total:	100	

Table S4.- Experimental working conditions of FESEM-EDX.

Working conditions<br>A FESEM Zeiss (Orsay Physics Kleindiek Oxford Instruments) model Auriga compact equipment was used for the FESEM-EDX examination. The X-ray microanalysis was performed using an Oxford-X Max X-ray microanalysis system coupled to the FESEM.<br>Operation conditions in the FESEM-EDX: A voltage of 20 kV. a current beam of 3.76-3.83 μA. resolution of 127 eV at 5.9 keV. X-ray detector operates at a working distance of 6-7 mm. The electron beam was always disposed perpendicularly to the polished surface of the cross-sections.<br>X-ray spectra acquisition conditions were: dead time 20 %. minimum number of counts acquired 2000. live time acquisition mode with an acquisition time of 100 s. process time of 5 s. number of channels automatically selected by the instrument that provided a channel width of 10 eV. Pulse pileup correction was active. which automatically performs deconvolution of overlapped peaks.<br>As samples are poor conductor. they were carbon coated for avoiding localized charging and any resulting distortion or reflection of the electron beam.<br>Software Aztec (Orsay Physics Kleindiek Oxford Instruments) has been used for controlling the acquisition of digital images and X-ray spectra.<br>Images were acquired with secondary and backscattered electron detector. A scansize of 1024. dwell time of 34 μs and frame time of 26.739 s were the image acquisition conditions.<br>The software Aztec automatically considers the C sputtering treatment and automatically re-calculates the intensity of the emission line of the C peak according to a calculated thickness of the external layer of C formed by the sputtering of 10 μm and density of 2.25 g cm^{-3}. Therefore. presence of C-containing minerals and organic matter was established from the rest of analytical techniques applied in this research.<br>Certificate reference standard materials used for calibrating the instrument:<br>C: $CaCO_3$; O: SiO_2; Na: Albite; Mg: MgO; Al: Al_2O_3; Si: SiO_2; P: GaP; S: FeS_2; Cl: KCl; K: MAD-10 Feldspar; Ca: wollastonite; Ti: Ti; Mn: Mn; Fe: Fe; Cu: Cu; Pb: PbF_2; Co: Co; Zn: Zn; Ni: Ni; Sn: Sn.
Qualitative measurements:<br>LOD: a theoretical averaged LOD for SEM–EDS measurements has been established in 0.08 wt% (Reed. 1996). Nevertheless. a common procedure for a more accurate calculation of the LODs in the FESEM and SEM from experimental measurements procedures (Veritá et al. 1994). According to this last method the averaged LOD value calculated from the experimental X-ray spectra obtained for the set of elements analyzed are in the range 0.07 - 0.1 wt%.
Quantitative measurements:<br>The standard deviation of the wt% values of the different elements is calculated by the Aztec software after applying the ZAF method of correction of interelemental effects on the intensity values for each element in each X-ray spectrum. Values obtained are in good agreement with those reported in similar studies of archaeological glass and ceramic materials (Kuisma-Kursula. 2000).<br>Accuracy of the FESEM-EDX instrument and applied method is calculated from NIST clay standard reference material (SRM 679-Brick clay) and NIST glass standard reference material (SRM 620-soda-lime glass) for considering the effect of the state of the sample (irregular and multi-mineralogical phase powder or flat and homogeneous amorphous material) in the accuracy provided by the analytical method. The values provided in the table thereafter correspond to the relative error. expressed in percentage ((experimental observed composition – certified composition) x)/certified composition)) x100%. Results obtained are similar to those reported by other authors previously (Kuisma-Kursula. 2000).

**NIST brick-clay SRM 679**<br>**Brick clay powder**	**NIST Glass SRM 620**<br>**Soda-lime glass platelets (35x35x3) mm**

Element	certified (wt %)	RD (%)	Oxide	certified (oxide wt %)	RD (%)
Al	11.01	3	SiO_2	72.08	2
Ba	0.0432	145	Al_2O_3	1.80	5
Ca	0.1628	45	Fe_2O_3	0.043	87
Ce	105*	n.d.	TiO_2	0.018	110
Cs	9.2*	n.d.	CaO	7.11	2
Cr	109.7*	n.d.	MgO	3.69	3
Co	26*	n.d.	K_2O	0.41	6
Cu	1.9*	n.d.	Na_2O	14.39	2
Hf	4.6*	n.d.	As_2O_3	0.056	75
Li	71.7*	n.d.			
Mg	0.7552	5			
Mn	1730*	9			
P	0.075	46			
K	2.43	7			
Rb	190*	n.d.			
Sc	22.5*	n.d.			
Si	24.34	2			
Na	0.1304	4			
Sr	73.4*	n.d.			
Th	14*	n.d.			
Ti	0.577	5			
Zn	150*	n.d.			
Fe	9.05	3			
* value is in mg kg^{-1}					
n.d.: not detected					

References

Reed. S.J.B. (1996) Electron Microbe Analysis and Scanning Electron Microscopy in Geology. Cambridge University Press. Cambridge.

Veritá. M.; Basso. R.; Wypyski. M.T.; Koestler. R.J. (1994). X-ray microanalysis of ancient glassy materials: a comparative study of wavelength dispersive and energy dispersive techniques. Archaeometry. 36. 241-25.

Kuisma-Kursula. P. (2000). Accuracy. Precision and Detection Limits of SEM–WDS. SEM–EDS and PIXE in the Multi-Elemental Analysis of Medieval Glass. X-Ray Spectrometry. 29. 111–118.
